# Unlocking cardioprotection: iPSC exosomes deliver Nec-1 to target PARP1/AIFM1 axis, alleviating HF oxidative stress and mitochondrial dysfunction

**DOI:** 10.1186/s12967-024-05204-9

**Published:** 2024-07-26

**Authors:** Xiaobing Lv, Boqin Liu, Xiaoting Su, Xintao Tian, Huating Wang

**Affiliations:** 1 Department of Cardiology, Jinan Central Hospital, Shandong University, No.105 Jiefang Road, Lixia District, Jinan, Shandong Province 250013 P. R. China; 2 Department of Cardiology, the Affiliated Hospital of Qingdao University, Qingdao, 266000 P.R. China; 3 Department of Cardiology, Qingdao Municipal Hospital (West Yard), Qingdao, 266000 P.R. China; 4 Department of Obstetric, the Affiliated Hospital of Qingdao University, Qingdao, 266000 P.R. China; 5 Department of Emergency Internal Medicine, the Affiliated Hospital of Qingdao University, Qingdao, 266000 P.R. China

**Keywords:** Heart failure, Necrostatin-1, iPSCs-derived exosomes, Oxidative stress, Mitochondrial dysfunction, PARP1/AIFM1 axis

## Abstract

**Background:**

Heart failure (HF) is characterized by oxidative stress and mitochondrial dysfunction. This study investigates the therapeutic potential of Necrostatin-1 (Nec-1) delivered through exosomes derived from induced pluripotent stem cells (iPSCs) to address these pathologies in HF.

**Methods:**

An HF rat model was established, and comprehensive assessments were performed using echocardiography, hemodynamics, and ventricular mass index measurements. iPSCs were used to isolate exosomes, loaded with Nec-1, and characterized for efficient delivery into cardiomyocytes. The interaction between Nec-1-loaded exosomes (Nec-1-Exos), poly (ADP-ribose) polymerase 1 (PARP1), and apoptosis-inducing factor mitochondria-associated 1 (AIFM1) was explored. Gain-of-function experiments assessed changes in cardiomyocyte parameters, and histological analyses were conducted on myocardial tissues.

**Results:**

Cardiomyocytes successfully internalized Nec-1-loaded exosomes, leading to downregulation of PARP1, inhibition of AIFM1 nuclear translocation, increased ATP and superoxide dismutase levels, reduced reactive oxygen species and malonaldehyde levels, and restored mitochondrial membrane potential. Histological examinations confirmed the modulation of the PARP1/AIFM1 axis by Nec-1, mitigating HF.

**Conclusions:**

iPSC-derived exosomes carrying Nec-1 attenuate oxidative stress and mitochondrial dysfunction in HF by targeting the PARP1/AIFM1 axis. This study proposes a promising therapeutic strategy for HF management and highlights the potential of exosome-mediated drug delivery.

**Supplementary Information:**

The online version contains supplementary material available at 10.1186/s12967-024-05204-9.

## Introduction

Heart failure (HF) is a condition in which the heart fails to discharge its contents adequately [1]. HF is manifested with dyspnoea and fatigue, accompanied by typical physical signs, such as peripheral oedema, which produce cardiac structure impairment or dysfunction, resulting in ventricular filling or impaired ejection function [2, 3]. Despite treatment advances, there are a larger number of patients with HF who suffer impaired function, poor quality of life and premature death [4, 5]. It has been noted that mitochondrial dysfunction, a hallmark of HF, is characterized by impaired bioenergetics, oxidative stress and aldehydic load [6]. Mitochondria are considered double-membrane organelles in almost all eukaryotic cells [7]. Mitochondria are particularly vulnerable to oxidative damage, and damaged mitochondrial membrane is accompanied by the increase of oxidative stress, leading to the reduction of mitochondrial biogenesis [8]. Oxidative stress is also known to be related to HF [9]. Therefore, there is an urgent need to understand the pathogenesis of mitochondrial dysfunction and oxidative stress in HF and develop novel, effective therapeutic strategies for improving the outcome of patients with HF.

In 2006, Takahashi et al. proposed a scheme to dedifferentiate mature cells to produce human induced pluripotent stem cells (iPSCs), thus avoiding the ethical problems caused by the use of embryonic stem cells [10]. It has been demonstrated that iPSCs could differentiate into cells of all three germ layers, including cardiomyocytes [11]. In particular, iPSC is a promising treatment in regenerative therapy because these unique cells can be produced from any individual [12]. The roles of iPSC in treating HF have been reported [13]. Moreover, a recent study has confirmed that iPSC could derive exosomes, which serves as a therapeutic strategy for HF [14]. Exosomes are nano-sized vesicles involved in intercellular communication by carrying macromolecular substances, including proteins, lipids, and nucleic acids [15]. Exosomes can be released by various cell types, including stem cells and cancer cells [16]. It is interesting to note that exosomes derived from mesenchymal stem cells (MSCs) exert great effects on HF [17]. In addition, Necrostatin-1 (Nec-1) is reported to ameliorate HF [18]. Nec-1, a selective inhibitor of RIP1, could inhibit necroptosis by repressing receptor interacting-protein 1 (RIP1) kinase activity and RIP1–RIP3 interaction [19]. The protective roles of Nec-1 in intracerebral hemorrhage and spinal cord injury have been validated [20]. Zhang et al. have also revealed that Nec-1 could reduce infarct size to preserve cardiac function [21]. However, the mechanism by which iPSCs-derived exosome communication affects mitochondrial dysfunction and oxidative stress in HF involving Nec-1 is still poorly understood, highlighting a major gap in knowledge given that iPSCs-secreted exosome may be of significance to mitochondrial dysfunction and oxidative stress in HF. Hence, we hypothesized that the transfer of Nec-1 via iPSCs-exosomes might alter mitochondrial dysfunction and oxidative stress in HF.

## Materials and methods

### Ethics statement

The study protocol was approved by the Ethics Committee of the Affiliated Hospital of Qingdao University. All experimental animals operating procedures were in line with the standard of the *Guide for the Care and Use of Laboratory* animals published by National Institutes of Health (NO. QYFYWZLL26397). Efforts were made to minimize the pain of the experimental animals.

### Bioinformatics analysis

Necrostatin-1 target gene was obtained from CTD database. The HF, mitochondrial dysfunction, and oxidative stress-related genes (correlation score ≥ 5) were searched from GeneCards database, respectively. The intersection of the above four gene sets was taken to determine the important genes. The protein-protein interaction of the above key genes were analyzed using STRING database.

### Establishment of HF rat models

A total of 80 SPF healthy male Sprague–Dawley (SD) rats (aged 8 weeks) with an average weight of (230 ± 15) g were purchased from the Animal Experiment Center of the Affiliated Hospital of Qingdao University. They were raised in a temperature (22 ± 3℃), humidity (40–70%), and light (12-h light/12-h dark) controlled room。.

Rats were randomly divided into Normal (normal rats; *N* = 10), Sham (sham-operated rats; *N* = 10), and Model groups (HF rats; *N* = 60). HF rats were induced by the trans-ascending aortic constriction (TAC) procedure. Rats were aestheticized by 1% pentobarbital sodium (P3761, Sigma-Aldrich Chemical Company, St Louis, MO) via intraperitoneal injection. A 0.5 cm incision was made at the level of the chest, the chest was opened, and the thymus was retracted to reveal the aorta. A 4 − 0 silk suture was passed around the ascending aorta, and with a 25-gauge needle temporarily placed adjacent to the aorta, the suture was tied around both the aorta and needle. The needle was then removed, leaving the constricting suture around the aorta. Rats were routinely raised post-operation. Sham-operated rats underwent the same operation procedures, but only threading without ligation. At week 4 after operation echocardiography was performed with cardiac ultrasound. The success standard of HF was left-ventricular ejection fraction (LVEF) ≤ 45%.

Another 50 HF rats were injected with adeno-associated virus (AAV; 20 µL 3 × 10^11^ v.g/ml) and exosomes (200 ug; labeled by DiR), which were Nec-1 group (injection of Nec-1), blank-Exos, Nec-1-Exos, Nec-1-Exos + overexpression (oe)-negative control (NC), and Nec-1-Exos + oe-poly(ADP-ribose) polymerase 1 (PARP1), with 10 rats in each group. TAC surgery was performed after 3 days. HF rats were administered with exosomes via caudal vein injection once a week for 4 weeks after TAC surgery. After the experiment, mice were euthanized by cervical dislocation. The tissues such as the heart, liver, spleen, lungs, and kidneys of rats were collected and imaged using the IVIS Lumina II in vivo imaging system (PerkinElmer, Thermo Fisher Scientific, Waltham, MA) to detect the location of exosomes in different organs [22].

AAV was purchased from Shanghai Genechem Co., Ltd. (Shanghai, China). The silencing AAV was constructed with the GV478 vector, and the overexpression AAV was constructed with the GV388 vector of Shanghai Genechem. The primer sequence, vector construction, virus packaging and purification were provided by Shanghai Genechem. The experimental steps were carried out according to the instructions. The failed HF rat models were removed and supplemented.

### Echocardiography

Rats were anesthetized with an intraperitoneal injection of 1% pentobarbital sodium (P3761, Sigma-Aldrich). The chest was shaved. The rats were fixed in a supine position on a wooden board. The color ultrasonic diagnostic instrument (Vevo 2100, Visualsonics, Toronto, Canada) was used to measure the left-ventricular posterior wall dimension (LVPWD), interventricular septal dimension (IVSD), left-ventricular end-diastolic dimension (LVEDD), left-ventricular end-systolic diameter (LVESD), LVEF, and left-ventricular fraction shortening (FS), respectively.

### Hemodynamics

Rats were anesthetized with an intraperitoneal injection of 1% pentobarbital sodium. The rats were fixed in a supine position on the operating table. The multi-channel physiological signal acquisition system (MP150, BIOPAC, Goleta, CA) was utilized to measure left-ventricular systolic pressure (LVSP), left-ventricular end diastolic pressure (LVEDP), maximum left-ventricular pressure rise (+ dp/dt), and maximum left-ventricular pressure fall (-dp/dt).

### Detection of ventricular mass index

Following hemodynamics, the thoracic cavity of rats was opened, and the heart was quickly taken out and placed in the precooled HEPES solution. After washing, the right ventricle and aorta were cut open. After draining the water, the atrium and blood vessels were removed. Electronic balance was employed to measure the left-ventricular mass (LVM) and the right-ventricular mass (RVM). The ratios between the mass and weight of LVM and RVM were left ventricular mass index (LVMI) and right ventricular mass index (RVMI), respectively. After weighing, some tissues were fixed in 4% paraformaldehyde and embedded in conventional paraffin, and paraffin-embedded sections were prepared (4 μm), preserving at -80℃.

### Primary culture and transduction of cardiomyocytes

The ventricles of 2-day-old neonatal rats were taken under sterile conditions, washed with Hank’s solution at pH 7.2–7.4 for 3 times, and cut into pieces. The cells were separated by magnetic stirring in a sterile glass vial with collagenase II (1 mg/mL) (1148090, Sigma-Aldrich). After 90 min of differential adhesion, the isolated cells were placed in Dulbecco’s Modified Eagle’s Medium (DMEM; 10569044, Gibco, Grand Island, NY) containing 0.1 mM 5-bromo-2’-deoxyuridine (BrdU) (B5002, Sigma-Aldrich), 10% fetal bovine serum (FBS; 10,099,141, Gibco) and 1% penicillin-streptomycin (15,070,063, Gibco) to inhibit the proliferation of fibroblasts. After 48 h, the spontaneously contractile neonatal rat cardiomyocytes in HF rats were treated with DMEM containing 10 umol/L Angiotensin-II (Ang II) (23–0101, Sigma-Aldrich) for 72 h to induce cardiomyocyte hypertrophy. The cells in normal rats were cultured in a 37℃ 5% CO_2_ incubator without any treatment. In order to detect the success of Ang II-induced cardiomyocyte hypertrophy, the cells were fixed with 4% paraformaldehyde for 10 min, washed with phosphate buffer containing 0.1% Triton X-100, blocked with 3% BSA, and stained with phalloidin-FITC (Actin-tracker green) (C1033, Beyotime Institute of Biotechnology, Shanghai, China) for 60 min and Hoechst 33,258 (H1398, Thermo Fisher Scientific) for 15 min. The green fluorescence of microfilaments was observed under the confocal microscope, and the area of cardiomyocytes was measured by a micrometer.

The cells were transduced with oe-NC, oe-PARP1, blank-Exos, Nec-1-Exos, Nec-1-Exos + oe-NC, and Nec-1-Exos + oe-PARP1. The cells were seeded on the 6-well plates at a density of 5 × 10^5^ cells/well. Upon cell confluency reached 50–70%, cells were transduced with 40 µL/mL lentivirus for 6 h with the virus titer of 1 × 10^9^ TU/mL. The solution was changed and the culture was continued. The core plasmid (Fugw-GFP, Plx304) and auxiliary plasmid (RRE, REV, Vsvg) inserted with the cDNA sequence of the target gene were used to package the overexpressed lentivirus. The lentivirus was purchased from Shanghai Sangon Biotechnology Co. Ltd., (Shanghai, China). The primer sequence and plasmid construction were completed by Shanghai Sangon.

### Culture of iPSCs

The human iPSC line was obtained from The National Stem Cell Resource Center and East China Stem Cell Center. This cell line was generated from human dermal fibroblasts and obtained by reprogramming HEF cells with a non-integrated plasmid. The cell line was stably sub-cultured to 40 passages, and the characteristics of iPSCs remained intact. The markers of iPSCs were identified. AKP, Oct4, Sox2, Nanog, and Tra-1-60 were positively expressed. Teratoma formation was normal, and there was no integration in the identification of genomic pluripotency gene integration. When the confluency reached 80–90%, the iPSCs were dissociated by treatment with 0.5 mM EDTA and plated onto a truncated human vitronectin-coated culture dish. The cells were cultured in Essential 8 medium (Thermo Fisher Scientific) and passaged every 4 to 5 days. All cells were cultured at 37 °C in a humidified atmosphere containing 5% CO_2_.

### Isolation and identification of exosomes

The cell culture medium containing exosomes was centrifuged at 300 × g for 5 min to remove cells and then centrifuged at 10,000 g for 30 min to remove dead cells and cell debris. Finally, cells were centrifuged at 100,000 × g for 90 min using the L-100 XP ultracentrifuge (XPN-100, Beckman Coulter, Chaska, MN). After removal of the supernatant, the exosomal pellet was resuspended in 1 mL of 1 × phosphate-buffered saline (PBS). Further ultracentrifuge was performed at 100,000 × g for 70 min to remove the remaining medium components in the exosomes. Finally, the exosomes were resuspended in 100 µL 1 × PBS and stored at 80 °C. All centrifugation steps were performed at 4 °C.

Transmission electron microscopy (TEM) was performed to identify exosomes. In brief, 30 µL exosomes were dropped on a copper mesh, standing for 1 min. The liquid was sucked up from the side with a filter paper. The exosomes were counterstained with 30 µL phosphotungstic acid solution (pH 6.8) at room temperature for 5 min, dried in an incandescent lamp, observed and photographed under a Tecnai T10 TEM (FEI, Blackwood, NJ).

Dynamic Light Scattering (DLS) was used to detect the diameter of exosomes. The Zetasizer Nano-ZS90 instrument (Malvern Panalytical Ltd., Marvin, UK) with excitation light wavelength λ = 532 nm was utilized for the experiment. The exosomes were diluted with 0.15 M NaCl to an appropriate optical signal detection level (the ratio was 1: 50), and the detection was performed after mixing.

The concentration of exosomal protein was determined by bicinchoninic acid (BCA) protein analysis kits (P0011, Beyotime). Western blot analysis was adopted to detect the expression of exosome-related proteins (CD9, CD63 and TSG101).

### Exosomes loaded with Nec-1

To load engineered exosomes with exogenous cargo, Nec-1 (HY-15,760, MedChemExpress, Monmouth Junction, NJ) was transfected by Neon electroporation (1000 V, 10 ms, 2 pulses). Exosomes at a total protein concentration of 10 µg (measured by BCA) were mixed with 10 µg Nec-1 in R buffer from the Neon kit (Invitrogen Inc., Carlsbad, CA) before electroporation. After electroporation, exosomes were washed two times in PBS with 100,000 × g centrifugation.

To calculate the cargo loading efficiency, after electroporation treatment, samples were diluted with 100 × PBS and centrifuged at 100,000 × g for 70 min to remove free Nec-1. On the other hand, a spectrophotometer was employed to determine the envelopment rate of Nec-1. Nec-1-Exos (50 mg) were distributed in 50 ml of 1 mol/L HCl by sonication for 1 h. The concentration of Nec-1 in the supernatant was assayed by a UV–Vis spectrophotometer (UV–Vis 8500, Techcomp, Shanghai, China) at 265 nm and the supernatant from vacuous exosomes was used as a contrast. The drug loading capacity of Nec-1-Exos was calculated by the following formulas: LC% = W1/W2 × 100%, where W1 is the weight of Nec-1 encapsulated in the exosomes, and W2 is the gross weight of the Nec-1-Exo. Under the optimum electroporation conditions, HPLC results showed that the Nec-1 loading (LC) of exosomes was 3.1%.

### Tracing of exosomes in vitro

The isolated exosomal membranes were stained with PKH67 Green Fluorescent Cell Ligase Mini Kit (Mini67-1KT, Sigma-Aldrich). All staining steps were carried out in accordance with the instructions. The exosomes were mixed with a dyed solution, incubated at 37°C for 15 min, and centrifuged at 120,000 × g for 90 min by ultracentrifugation to remove the non-binding dye. After two PBS washes, the exosomes were centrifuged at 120,000 × g for 90 min, and the labeled exosomes were resuspended in PBS. The fluorescence-labeled exosomes were added to the cell supernatant. After incubation for 48 h, the supernatant was discarded, the excessive exosomes were washed away with PBS, and then the nucleus was stained with 4’,6-diamidino-2-phenylindole (DAPI) for 5 min. The fluorescence signal of the cells was detected by a laser confocal microscope (FV1000, Olympus Optical Co., Ltd, Tokyo, Japan) to determine whether the added exosomes and cells were fused.

### Reverse transcription quantitative polymerase chain reaction (RT-qPCR)

Trizol (16,096,020, Thermo Fisher Scientific) was used to extract total RNA from cells, and the NanoDrop One/OneC micro-nucleic acid protein concentration analyzer (Thermo Fisher Scientific) was utilized to detect RNA concentration and purity with A260/A280 = 2.0 and the concentration of more than 5 µg/µL. mRNA was reverse-transcribed into cDNA using the cDNA first-strand synthesis kit (D7168L, Beyotime) according to the instructions. RT-qPCR was performed using RT-qPCR kits (Q511-02, NanJing Vazyme Biotech Co., Ltd., Nanjing, China). PCR amplification was conducted on the Bio-rad real-time qPCR instrument CFX96 (Bio-rad, Hercules, CA). GAPDH served as an internal reference, and the primer sequences (Table S1) were designed and provided by Shanghai Sangon. The 2^−ΔΔCt^ method was used to quantify the relative expression of target genes.

### Western blot analysis

Total protein was extracted from tissues, cells, or exosomes using radio immunoprecipitation assay (RIPA) lysis buffer (P0013B, Beyotime) containing 1 mM PMSF. Nuclear and cytoplasmic proteins were extracted using the nuclear and cytoplasmic separation kit (P0028, Beyotime). The protein concentration was measured using the BCA Kit (P0011, Beyotime) and adjusted to 1 µg/µL. The sample in each tube (100 µL) was boiled at 100 °C for 10 min to denature the protein and stored at -80 °C. The proteins (10 µL) were centrifuged, subjected to 8-12% sodium dodecyl sulfate-polyacrylamide gel electrophoresis gels (SDS-PAGE), electrotransferred onto a polyvinylidene fluoride (PVDF) (1,620,177, Bio-Rad). The membrane was blocked with 5% bovine serum albumin (BSA) at room temperature for 1 h, and then incubated with the prepared rabbit antibodies GAPDH (ab181602, 1: 5000, Abcam, Cambridge, UK), Collagen I (ab270993, 1: 1000, Abcam), Collagen III (ab7778, 1: 1000, Abcam), COX IV (ab202554, 1: 1000, Abcam), Histone H3 (ab215728, 1: 1000, Abcam), apoptosis inducing factor mitochondria associated 1 (AIFM1) (ab1998, 1: 1000, Abcam), PARP1 (ab191217, 1: 1000, Abcam), RIPK1 (17519-1-AP, 1: 1000, Proteintech), RIPK3 (17563-1-AP, 1: 1000, Proteintech), CD9 (ab92726, 1: 1000, Abcam), CD63 (ab134045, 1: 1000, Abcam), TSG101 (ab125011, 1: 1000, Abcam), and Calnexin (ab133615, 1: 1000, Abcam) at 4 °C overnight. The next day, the membrane was incubated with the horseradish peroxidase (HRP)-labeled secondary antibody goat anti-rabbit IgG (ab6721, 1: 5000, Abcam) at room temperature for 1 h and developed with chemiluminescence (ECL) working solution (1,705,062, Bio-Rad) for 1 min. The liquid was aspirated, and the membrane was covered with plastic wrap. The protein bands were exposed for imaging on the Image Quant LAS 4000 C gel imager (GE Healthcare, Milwaukee, WI). The relative protein expression was expressed as the ratio of the gray value of protein to be tested to that of internal reference (GAPDH).

### Immunofluorescence staining

The treated cells were collected and fixed with 4% paraformaldehyde for 15 min. After being blocked with 5% goat serum (diluted with 0.3% PBST) at room temperature for 1 h, cells were incubated with diluted primary antibody, AIFM1 (ab1998, 1: 200, Abcam) at room temperature for 1 h. Afterward, cells were incubated with a red fluorescent protein (Cy3)-labeled secondary antibody goat anti-rabbit (ab6939, 1: 500, Abcam) at room temperature for 1 h. At last, the slides were sealed with mounting tablets containing DAPI, dried, and photographed under a microscope (BX63, Olympus).

### Superoxide dismutase (SOD) activity and malondialdehyde (MDA) level

The SOD level in the heart tissues and cardiomyocytes of rats was measured using the SOD Kit (S0101S, Beyotime). About 1 × 10^6^ cells or 10 mg tissues were added to 100 µL SOD sample preparation solution, pipetted or homogenized appropriately to fully lyse the cells, followed by centrifugation at about 12,000 g at 4 °C for 3–5 min to obtain the supernatant. An appropriate amount of WST-8 enzyme working solution was prepared with a volume of 160 µL and incubated for 30 min, and the absorbance at the wavelength of 450 nm was measured to calculate the SOD activity. The content of MDA was detected by MDA kits (S0131S, Beyotime) according to the instructions. Tissues or cells were lysed or homogenized, and centrifuged at 10,000 g-12,000 g for 10 min. TBA storage solution was prepared, and the reaction system was tested according to the instructions. The samples were mixed with 0.1 mL sample and 0.2 mL MDA detection working solution and boiled for 15 min. The samples were cooled to room temperature in a water bath and centrifuged at 1000 g for 10 min at room temperature. The supernatant (200 µL) was added to a 96-well plate, and the absorbance was measured at the wavelength of 532 nm with a microplate reader to calculate the MDA content.

### Mitochondria extraction

We used the mitochondria extraction kit (tissues) (C3606, Beyotime) to extract mitochondria from tissues according to the manufacturer’s instructions. In brief, 80 mg of myocardial tissue was cut completely and washed in PBS three times in a 1.5-mL tube. Then, 640 µL A liquor and two beads were added to the tube. The tube was placed into a lapping machine for 20 s. The tube was centrifuged at 600 × g for 5 min, and the supernatant was transferred to a new tube, followed by centrifugation at 11,000 × g for 10 min. The supernatant was cytoplasmic protein. Mitochondria were extracted using the mitochondria extraction kit (cell) (C3601, Beyotime) according to the manufacturer’s instructions. Briefly, 2 × 10^7^ cells were detached with 0.25% trypsin, centrifuged at 100 g for 5–10 min and ground in a lapping machine for 20 s. The tube was centrifuged at 600 × g for 5 min. The supernatant was transferred to a new tube, followed by centrifugation at 11,000 × g for 10 min. The precipitate contained mitochondria. The supernatant was carefully removed, collected, and centrifuged at 12,000 g for 10 min at 4 °C. The supernatant was the cytoplasmic protein.

### Adenosine triphosphate (ATP) content

The intracellular ATP content was determined using an enhanced ATP determination kit (S0026, Beyotime). In short, the cells were lysed with ATP lysis buffer and centrifuged at 12,000 g for 10 min at 4 °C. The supernatant was removed and mixed with the diluent containing luciferase. According to the instructions, the relative light unit was measured with a Multiskan FC spectrophotometer (51,119,000, Thermo Fisher Scientific). A fresh standard curve was made each time to calculate the ATP content.

### Mitochondrial reactive oxygen species (ROS) generation

Mito-SOX (M36008, Thermo Fisher Scientific) was used to detect the production of ROS in cells. The cells were incubated with 1 mL 5 μm Mito-SOX at 37 °C for 10 min, stained with Hoechst 33,342 (62,249, 1: 500, Thermo Fisher Scientific) for 10 min, and fixed in 4% paraformaldehyde for 15 min at 4℃. Finally, the cells were observed under a microscope (BX63, Olympus), and the red fluorescence density was calculated by Image-Pro Plus 6.0 software.

### Measurement of mitochondrial membrane potential

JC-1 Mitochondrial Membrane Potential Measurement Kit (C2006, Beyotime) was utilized to detect changes in mitochondrial membrane potential. When the mitochondrial membrane potential was high, JC-1 was gathered in the matrix of the mitochondria to form aggregates. When the mitochondrial membrane potential was low, JC-1 could not accumulate in the matrix of the mitochondria; at this time, JC-1 was a single body (monomeric). After different treatments, 1 mL JC-1 staining solution was added to incubate the cells at 37 °C for 20 min. The cells were collected, and tested with a flow cytometer (Becton Dickinson, Franklin Lakes, NJ).

### Flow cytometry for apoptosis of cardiomyocytes

After transduction for 48 h, the cells were detached with 0.25% trypsin (without EDTA) and collected in a flow tube. The Annexin V-FITC and propidium iodide (PI) kits (C1062L, Beyotime) were used to detect cell apoptosis. After different treatments, the cells were cultured in an incubator for 48 h. The cells were collected in 200 µL buffer solution, and 10 µL Annexin V-FITC and 5 µL PI were added to react for 15 min at room temperature, avoiding light exposure, followed by the addition of 300 µL buffer. The flow cytometer (Becton Dickinson) was utilized to detect cell apoptosis and the rate of apoptosis was counted.

### Terminal deoxyribonucleotidyl transferase (TdT)-mediated biotin-16-dUTP nick-end labelling (TUNEL) assay for apoptosis in heart tissues

The cell apoptosis detection kit (C1098, Beyotime) was employed to detect apoptosis in heart tissues. The paraffin-embedded sections were deparaffinized and hydrated. The sections were incubated with 20 µg/ml proteinase K without DNase at 37 °C for 15 min. Next, the sections were incubated with TUNEL detection solution (50 µL) at 37 °C for 60 min in the dark, mounted with DAPI, and observed and photographed under a microscope (BX63, Olympus). The apoptosis rate was counted.

### Masson staining

After dewaxing and hydration, the sections were immersed in 10% trichloroacetic acid and 10% potassium dichromate liquids for 40 min, rinsed in tap water, and stained with hematoxylin (PT001, Shanghai Bogoo Biotechnology Co., Ltd., Shanghai, China) for 8 min. Subsequently, the sections were immersed in 1% Ponceau (HL12202, Shanghai Haring Biological Technology Co., Ltd., Shanghai, China) and 1% Magenta mixture (HPBIO-SJ820, Shanghai Hepeng Biological Technology Co., Ltd., Shanghai, China) for 40 min. The 1% glacial acetic acid and 1% molybdic acid solution were successively added to terminate the reaction. The sections were mounted with neutral gum, observed and photographed under a microscope (BX63, Olympus). Five visual fields were randomly selected for each section. Image-Pro Plus 6.0 software was used to calculate myocardial collagen volume fraction (CVF) with CVF (%) = collagen area / full visual field area × 100%.

### Detection of intracellular Nec-1 levels by LC-MS/MS

In accordance with previous research, LC-MS/MS was employed to quantify Nec-1 content in cardiomyocytes. Briefly, rat cardiac myocytes were disrupted, placed in a centrifuge tube, and mixed with 5 µL of 1 µg/mL internal standard diazepam (d0940000, Merck). After extraction with 200 µL of acetonitrile (34,851, Merck), the mixture was vigorously vortexed for 2 min, incubated at 4 °C for 20 min, and centrifuged at 18,000 g for 20 min. The supernatant was transferred to another tube, evaporated to dryness under a gentle nitrogen stream at room temperature, and reconstituted with mobile phase (water: acetonitrile = 1:1 with 0.1% formic acid, 100 µL). Subsequently, aliquots of the solution (2 µL) were injected into the HPLC-MS/MS system for analysis [23].

### Co-IP

Cardiomyocytes were lysed using RIPA buffer, followed by a 2-hour incubation at room temperature with either anti-IgG (ab182016, Abcam) or anti-PARP1 (ab227244, Abcam) antibodies. Subsequently, the incubation mixture was further incubated with Protein A/G beads for 1 h. The target protein abundance was assessed by Western blotting after washing and resuspending in RIPA buffer [24].

### Statistical analysis

All data were summarized as mean ± standard deviation and analyzed by SPSS 21.0 statistical software (IBM Corp., Armonk, NY). Comparisons of data between the two groups were analyzed using an independent-sample *t*-test. A one-way analysis of variance (ANOVA) with Tukey’s post-hoc test was applied to compare of data among multiple groups. A value of *p* < 0.05 was statistically significant.

## Results

### Successful establishment of HF rat and cell models

In order to verify the effect of iPSCs-exosomes loaded with Nec-1 on HF rats, the HF rat and cell models were constructed. Ultrasound diagnostic instruments showed that IVSD, LVEDD, LVESD, and LVPWD increased, while LVEF and FS were reduced in HF rats (Table S2).

The results of hemodynamics exhibited that LVEDP increased, LVSP, +dp/dt, and -dp/dt decreased in HF rats (Table S3).

**Fig. 1:**
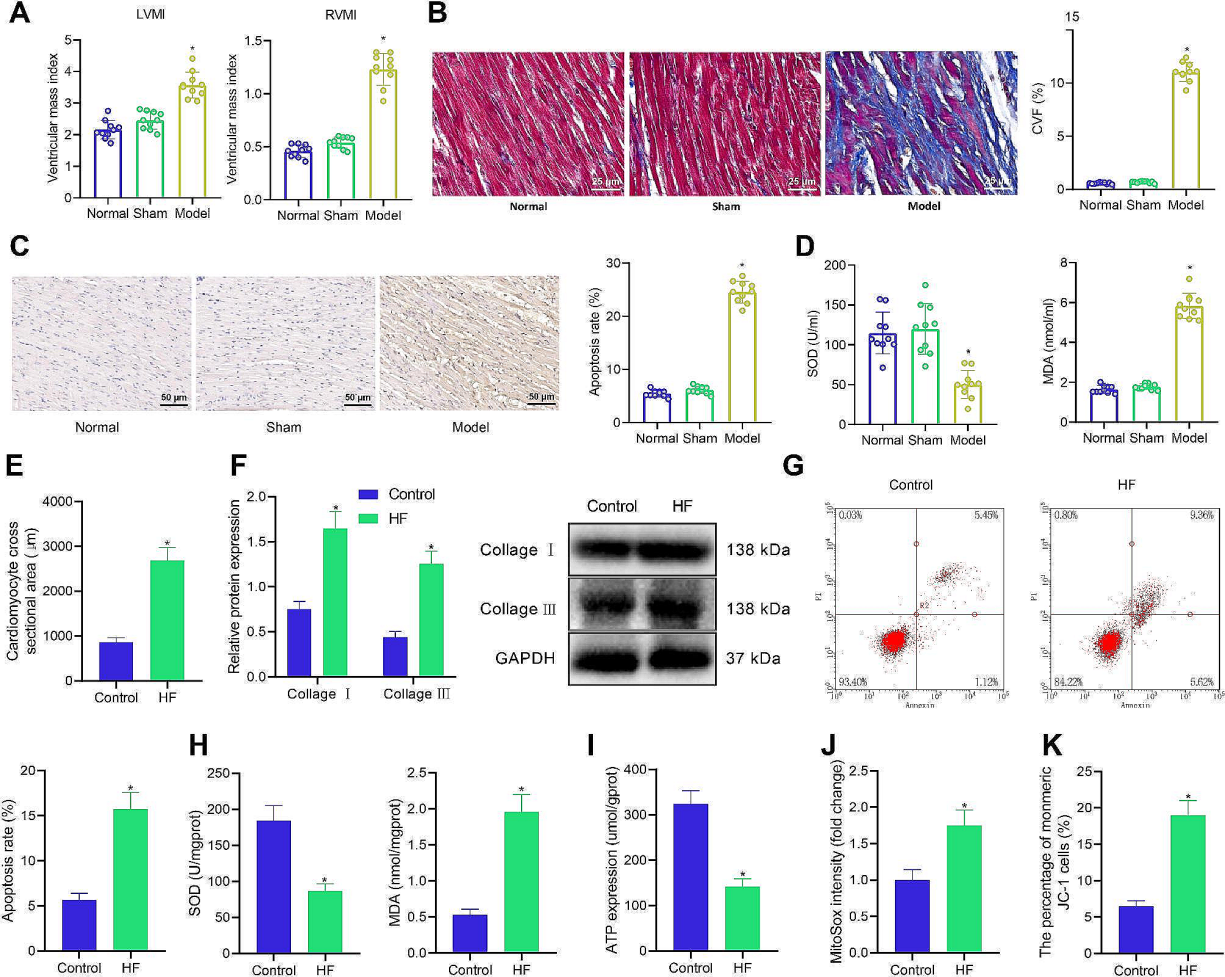
Establishment of HF rat and cell models. **A**, Changes of ventricular mass index in HF rats. **B**, Collagen fibers in myocardial tissues of HF rats detected using Masson staining. **C**, Apoptosis in myocardial tissues of HF rats detected using TUNEL staining. **D**, Measurement of SOD activity and MDA level in HF rats. **E**, Cardiomyocyte hypertrophy of HF cardiomyocytes detected using phalloidin-FITC staining. **F**, Protein levels of Collagen I and Collagen III in HF cardiomyocytes determined using Western blot analysis. **G**, Apoptosis of HF cardiomyocytes detected by flow cytometry. **H**, Measurement of SOD activity and MDA level in HF cardiomyocytes. **I**, Detection of mitochondrial ATP generation ability in HF cardiomyocytes. **J**, Mitochondrial ROS generation ability in HF cardiomyocytes detected by Mito-SOX. **K**, The changes in mitochondrial membrane potential detected by flow cytometry. * *p* < 0.05 vs. sham-operated rats or normal cardiomyocytes. *n* = 10. Cell experiments were repeated three times

The results of the ventricular mass index test and Masson staining also found that LVMI, RVMI, and CVF of HF rats were elevated (Fig. 1A, B).

TUNEL staining displayed that the apoptosis of cardiomyocytes was promoted in HF rats (Fig. 1C). Antioxidant index test showed that SOD activity was reduced, and the MDA level increased in the heart tissues of HF rats (Fig. 1D).

Based on the verification results in the HF cell models, the phalloidin-FITC staining and Western blot assay showed that the cross-sectional area, Collagen I, and Collagen III in HF cardiomyocytes increased (Fig. 1E, F). Flow cytometry revealed that apoptosis of HF cardiomyocytes was enhanced (Fig. 1G). It was also found that SOD activity, ATP level, and membrane potential decreased, and MDA level and ROS generation increased in HF cardiomyocytes (Fig. 1H-K).

These results suggested that the HF rat and cell models were successfully constructed.

### The successful construction of exosomes loaded with Nec-1

**Fig. 2:**
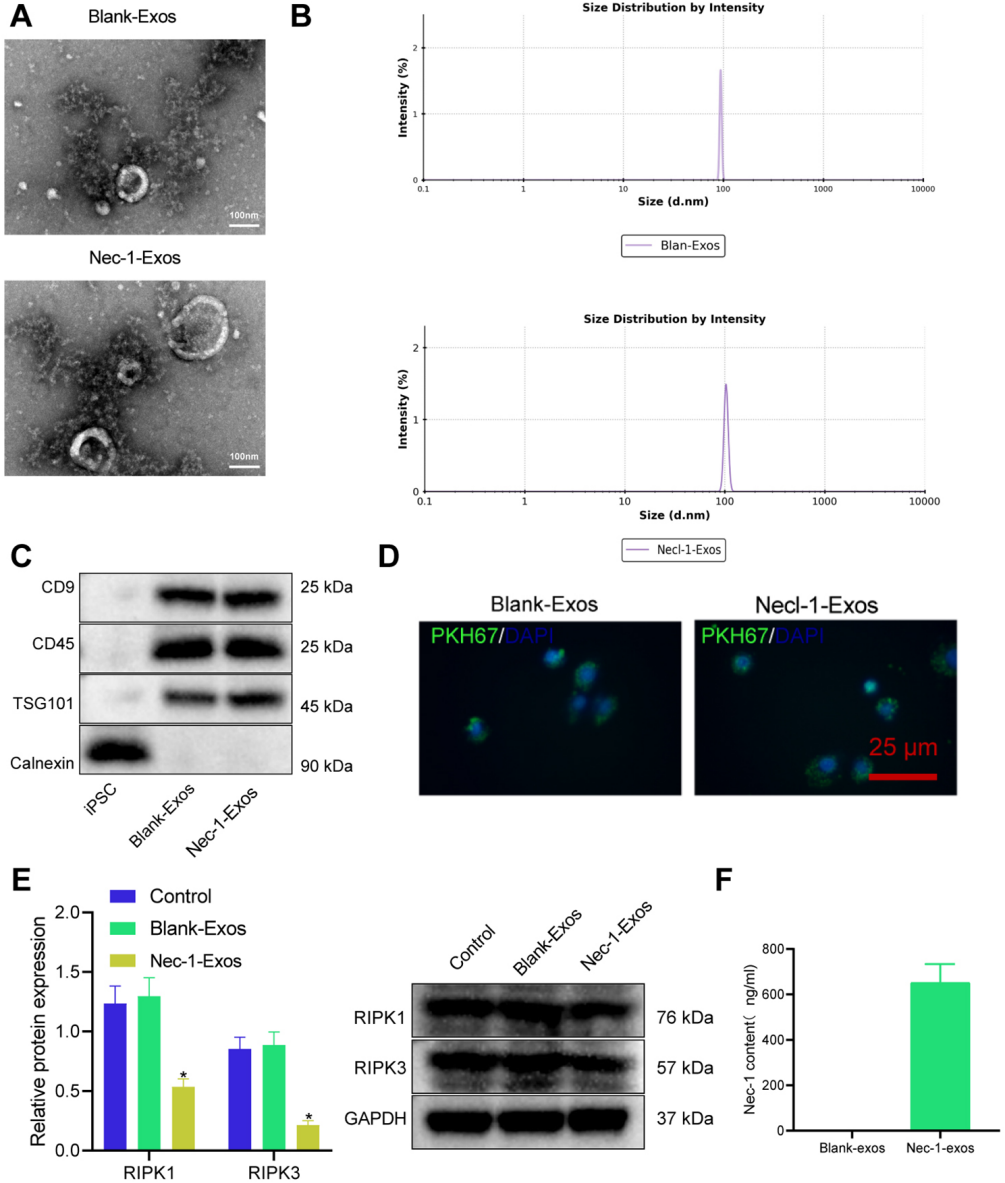
Isolation and identification of exosomes-loaded Nec-1. **A**, The structure of exosomes observed under the TEM. **B**, The size of exosomes detected using DLS. **C**, Exosomes-related protein bands detected using Western blot analysis. **D**, Uptake of exosomes by cardiomyocytes observed under a fluorescence microscope. **E**, Protein levels of RIPK1, RIPK3, and Nec-1 in HF cardiomyocytes treated with Nec-1-Exos. **F**, LC-MS/MS detection of Nec-1 content in cardiomyocytes. * *p* < 0.05 vs. HF cardiomyocytes treated with blank-Exos. Cell experiments were repeated three times

Nec-1 was introduced into iPSC-derived exosomes using electroporation. First, we identified iPSC-derived exosomes and observed the saucer-like three-dimensional structure of the exosomes with clear membranes under a TEM (Fig. 2A). The size of exosomes varied from 30 nm to 200 nm by DLS (Fig. 2B). Western blot analysis revealed that the levels of related proteins CD9, CD63, and TSG101 increased in exosomes, but Calnexin was not expressed (Fig. 2C). These results indicated that the exosomes were successfully extracted.

The uptake of exosomes by cardiomyocytes was observed under a confocal fluorescence microscope. The results showed that the uptake of PKH67-Exos by cardiomyocytes was obvious at 48 h of co-culture (Fig. 2D), indicating that exosomes could be transferred to cardiomyocytes. Nec-1, as a RIP1 kinase inhibitor, could inhibit the expression of RIPK1 and RIPK3 proteins. Western blot analysis presented that the Nec-1-Exos could significantly reduce the protein levels of RIPK1 and RIPK3 (Fig. 2E). The LC-MS/MS analysis revealed a significant increase in the concentration of Nec-1 in cardiomyocytes of the Nec-1-exos group compared to the Blank-exos group, indicating successful intracellular delivery of Nec-1 to the cardiomyocytes (Fig. 2F). It can be speculated that isolated exosomes could be loaded with Nec-1.

### Nec-1-Exos inhibit oxidative stress and mitochondrial dysfunction in cardiomyocytes

**Fig. 3:**
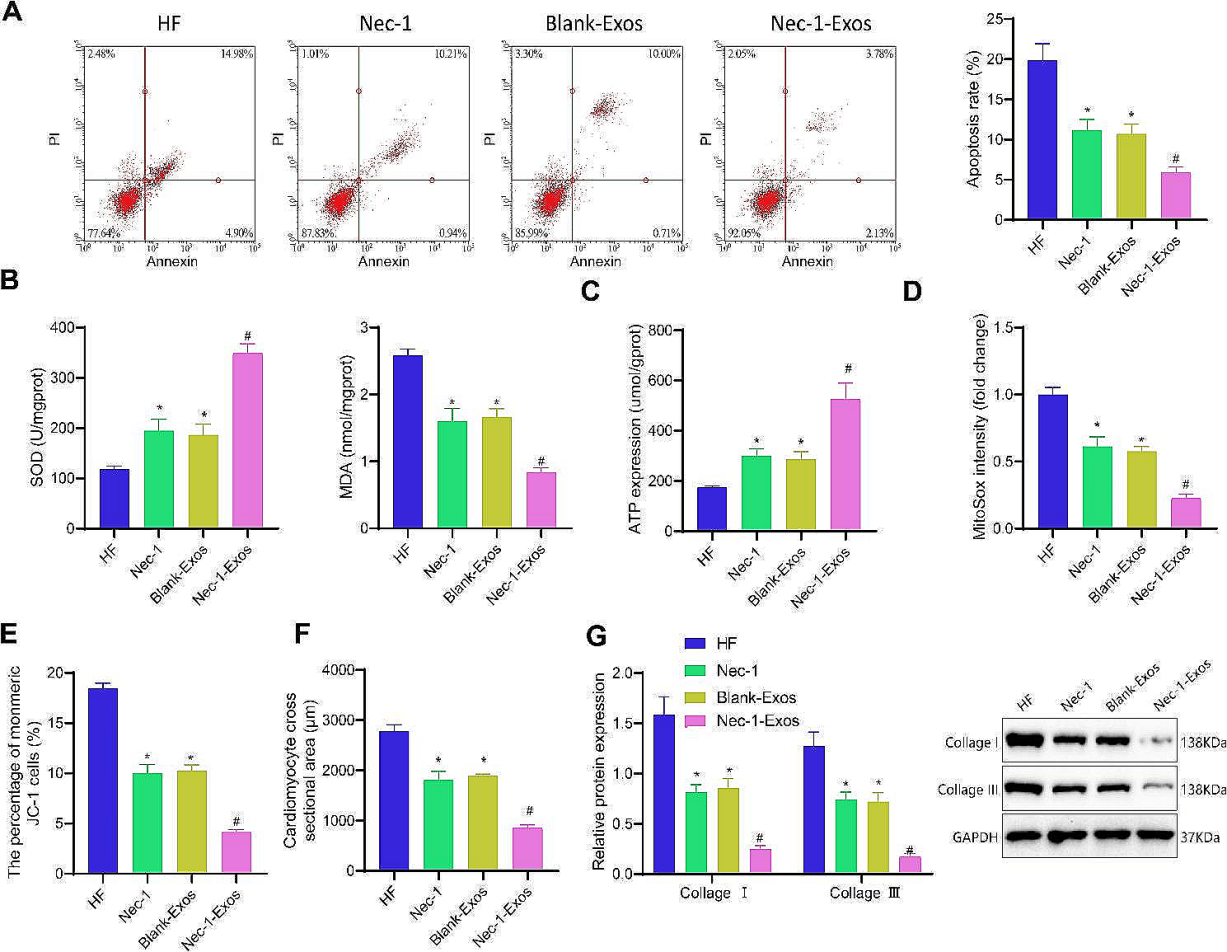
Effects of Nec-1-Exos on oxidative stress and mitochondrial dysfunction of cardiomyocytes. HF cardiomyocytes were treated with blank-Exos or Nec-1-Exos. **A**, Apoptosis of HF cardiomyocytes detected by flow cytometry. **B**, Measurement of SOD activity and MDA level in HF cardiomyocytes. **C**, Detection of mitochondrial ATP generation ability in HF cardiomyocytes. **D**, Mitochondrial ROS generation ability in HF cardiomyocytes detected by Mito-SOX. **E**, The changes in mitochondrial membrane potential detected by flow cytometry. **F**, Cardiomyocyte hypertrophy of HF cardiomyocytes detected using phalloidin-FITC staining. **G**, Protein levels of Collagen I and Collagen III in HF cardiomyocytes determined using Western blot analysis. * *p* < 0.05 vs. HF cardiomyocytes without treatment; # *p* < 0.05 vs. HF cardiomyocytes treated with blank-Exos; & *p* < 0.05 vs. HF cardiomyocytes treated with Nec-1. Cell experiments were repeated three times

In further validating the impact of Nec-1-Exos on oxidative stress and mitochondrial dysfunction in cardiomyocytes, the results of flow cytometry indicated a significant decrease in apoptosis in the Nec-1 group and blank-Exos group compared to the HF group. Additionally, the Nec-1-Exos group showed a further significant decrease in apoptosis compared to the blank-Exos group, and a more pronounced decrease compared to the Nec-1 group (Fig. 3A). Antioxidant index measurements revealed that compared to the HF group, the SOD activity significantly increased and MDA levels decreased in the Nec-1 group and blank-Exos group. Moreover, compared to the blank-Exos group, the SOD activity further significantly increased and MDA levels further significantly decreased in the Nec-1-Exos group; and compared to the Nec-1 group, the SOD activity further significantly increased and MDA levels further significantly decreased in the Nec-1-Exos group (Fig. 3B). Mitochondrial function analysis showed that compared to the HF group, ATP levels and membrane potential significantly increased, while ROS levels significantly decreased in the mitochondria of the Nec-1 group and blank-Exos group. Furthermore, compared to the blank-Exos group, ATP levels and membrane potential further significantly increased, and ROS levels further significantly decreased in the Nec-1-Exos group. Likewise, compared to the Nec-1 group, ATP levels and membrane potential further significantly increased, and ROS levels further significantly decreased in the Nec-1-Exos group (Fig. 3C-E).

Phalloidin-FITC staining results revealed that compared to the HF group, the cross-sectional area of cells significantly decreased in the Nec-1 group and blank-Exos group. Notably, the cross-sectional area of cells in the Nec-1-Exos group further significantly decreased compared to the blank-Exos group, and compared to the Nec-1 group, it further significantly decreased in the Nec-1-Exos group (Fig. 3F). Western blot analysis of Collagen I and Collagen III demonstrated that compared to the HF group, the protein expression of Collagen I and Collagen III significantly decreased in the Nec-1 group and blank-Exos group. Furthermore, compared to the blank-Exos group, the protein expression of Collagen I and Collagen III further significantly decreased in the Nec-1-Exos group; and compared to the Nec-1 group, the protein expression of Collagen I and Collagen III further significantly decreased in the Nec-1-Exos group (Fig. 3G). These results indicate that Nec-1-Exos can inhibit oxidative stress and mitochondrial dysfunction in cardiomyocytes, and Exos loaded with Nec-1 exhibits superior effectiveness compared to Nec-1 treatment alone.

### Nec-1-Exos have a certain therapeutic effect on HF in rats

**Fig. 4:**
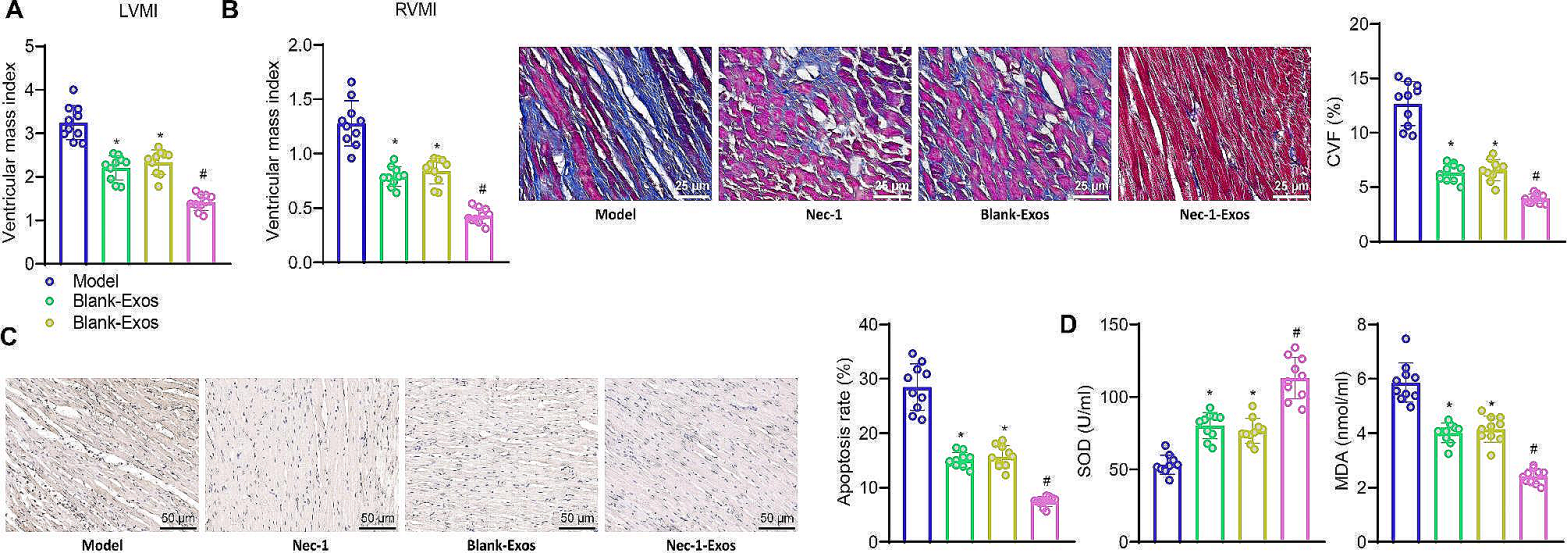
Effect of Nec-1-Exos on HF in Rats. HF rats were injected with blank-Exos or Nec-1-Exos (*n* = 10). **A**, Changes of ventricular mass index in HF rats. **B**, Collagen fibers in myocardial tissues of HF rats detected using Masson staining. **C**, Apoptosis in myocardial tissues of HF rats detected using TUNEL staining. **D**, Measurement of SOD activity and MDA level in HF rats. * *p* < 0.05 vs. HF rats; # *p* < 0.05 vs. HF rats injected with blank-Exos; & *p* < 0.05 vs. HF rats injected with Nec-1, *n* = 10

Nec-1-Exos could inhibit oxidative stress and mitochondrial dysfunction of cardiomyocytes in vitro, which was further verified in a rat model. We tested the distribution of Nec-1-Exos in various organs after tail vein injection, and the results showed that relative to the exosomes in those of HF rats injected with blank-Exos, the exosomes increased in the heart, lungs, kidneys, and colon but decreased in the liver and spleen of HF rats injected with Nec-1-Exos (Fig. S1). Ultrasound diagnostic instrument showed that IVSD, LVEDD, LVESD, and LVPWD decreased, while LVEF and FS increased in HF rats injected with Nec-1 or blank-Exos, and that IVSD, LVEDD, LVESD, and LVPWD significantly decreased, while LVEF and FS evidently increased in HF rats injected with Nec-1-Exos. Compared to the Nec-1 group, all parameters significantly improved in the Nec-1-Exos group (Table S4). Hemodynamic assessments revealed that compared to the Model group, the LVEDP significantly decreased in the Nec-1 group and blank-Exos group of rats, while the LVSP, maximum rate of rise of left ventricular pressure (+ dp/dt), and maximum rate of fall of left ventricular pressure (-dp/dt) significantly increased. Furthermore, in comparison to the blank-Exos group, all parameters further improved in the Nec-1-Exos group. Similarly, compared to the Nec-1 group, all parameters also significantly improved in the Nec-1-Exos group (Table S5). The results of LVMI and RVMI measurements showed a significant decrease in the Nec-1 group and blank-Exos group of rats compared to the Model group. Furthermore, the Nec-1-Exos group exhibited a further significant reduction in both LVMI and RVMI compared to the blank-Exos group, as well as a more pronounced decrease compared to the Nec-1 group (Fig. 4A).

Masson’s staining revealed that in comparison to the Model group, the collagen volume fraction (CVF) significantly decreased in the hearts of rats in the Nec-1 and blank-Exos groups. Moreover, the CVF in the hearts of rats in the Nec-1-Exos group further significantly decreased compared to the blank-Exos group, and a more pronounced decrease was observed compared to the Nec-1 group (Fig. 4B). TUNEL immunohistochemistry results demonstrated that apoptosis in myocardial tissue of rats was significantly reduced in the Nec-1 and blank-Exos groups compared to the Model group. Additionally, a further significant reduction in apoptosis was observed in the Nec-1-Exos group compared to the blank-Exos group, and a more pronounced decrease was noted compared to the Nec-1 group (Fig. 4C). Analysis of antioxidant parameters indicated that SOD activity significantly increased, and MDA levels significantly decreased in the hearts of rats in the Nec-1 and blank-Exos groups compared to the Model group. Furthermore, the SOD activity further significantly increased, and MDA levels further significantly decreased in the hearts of rats in the Nec-1-Exos group compared to the blank-Exos group, as well as compared to the Nec-1 group (Fig. 4D). These results suggest that Nec-1-Exos may have an inhibitory effect on heart failure in rats, and Exos loaded with Nec-1 exhibit better efficacy compared to Nec-1 treatment alone.

### Nec-1-Exos downregulate PARP1 in the nucleus and inhibit AIFM1 nuclear translocation

**Fig. 5:**
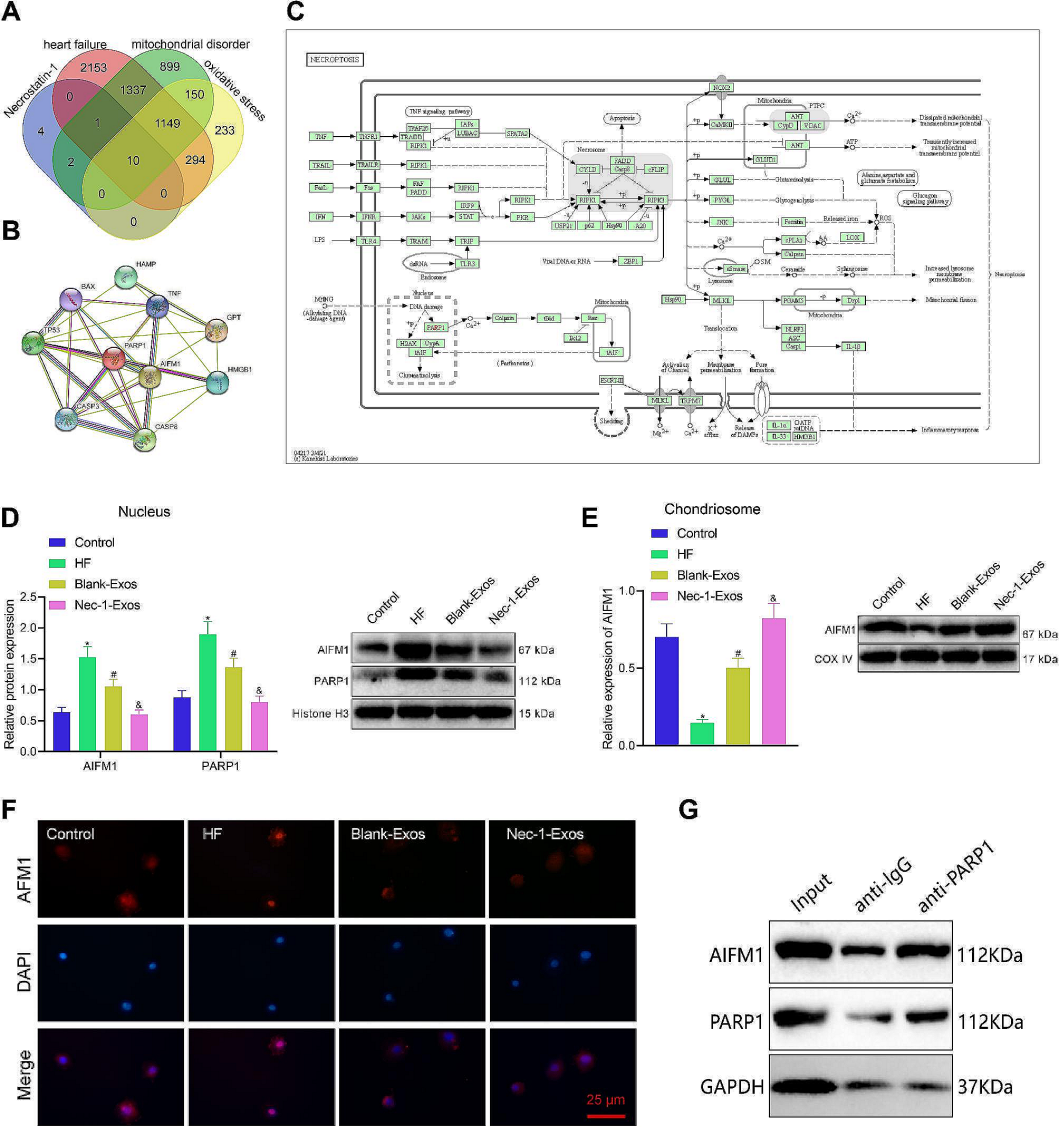
Nec-1-Exos affects PARP1/AIFM1 expression. **A**, Venn map of intersected genes between CTD and GeneCards database. **B**, Interactions of 10 key protein analyzed by STRING database. **C**, KEGG analysis of “necroptosis” signaling pathway (part). **D**, Protein levels of PARP1 and AIFM1 in nuclei of HF cardiomyocytes. **E**, AIFM1 protein level in mitochondria of HF cardiomyocytes. **F**, Nuclear translocation of AIFM1 in HF cardiomyocytes detected by immunofluorescence staining. **G**: Co-IP detection of the interaction between PARP1 and AIFM1 proteins. * *p* < 0.05 vs. normal cardiomyocytes; # *p* < 0.05 vs. HF cardiomyocytes without treatment; & *p* < 0.05 vs. HF cardiomyocytes treated with blank-Exos. Cell experiments were repeated three times

The target genes of Nec-1 were retrieved through the CTD database, and 17 genes were obtained. The HF, mitochondrial dysfunction, and oxidative stress-related genes were screened out through the GeneCards database with Relevance score ≥ 5 as the screening criteria, and there were 4944, 3548, and 1836 genes, respectively. The above results were intersected to get 10 more important genes, including HMGB1, AIFM1, CASP3, TNF, GPT, TP53, HAMP, CASP8, PARP1, BAX (Fig. 5A). STRING database exhibited that PARP1 and AIFM1 were in the key position of the regulatory network, with a regulatory relationship between them (Fig. 5B).

KEGG analysis showed that PARP1 in the “necroptosis” signaling pathway could promote the translocation of mitochondrial apoptosis-inducing factor AIFM1 (AIF) from mitochondria to the nucleus (Fig. 5C).

Western blot analysis displayed that expression of AIFM1 and PARP1 increased in the nucleus, and that AIFM1 expressed decreased in the mitochondria of HF cardiomyocytes, while the results were opposite in HF cardiomyocytes treated with blank-Exos. The expression of AIFM1 and PARP1 significantly decreased in the nucleus, and AIFM1 expression obviously increased in the mitochondria of HF cardiomyocytes treated with Nec-1-Exos (Fig. 5D, E). Immunofluorescence staining exhibited that the nuclear translocation of AIFM1 increased in the HF cardiomyocytes, which could be reduced after treatment with blank-Exos; however, the nuclear translocation of AIFM1 was not obvious in HF cardiomyocytes treated with Nec-1-Exos (Fig. 5F). Further Co-IP analysis revealed the presence of AIFM1 protein in the complexes pulled down by the PARP1 antibody. This finding indicates a physical interaction between PARP1 and AIFM1 (Fig. 5G).

These findings suggest that Nec-1-Exos may inhibit AIFM1 nuclear translocation by downregulating PARP1 expression in the nucleus and indicate a direct interaction between PARP1 and AIFM1.

### Nec-1-Exos inhibit oxidative stress and mitochondrial dysfunction in cardiomyocytes by inhibiting the PARP1/AIFM1 axis

**Fig. 6:**
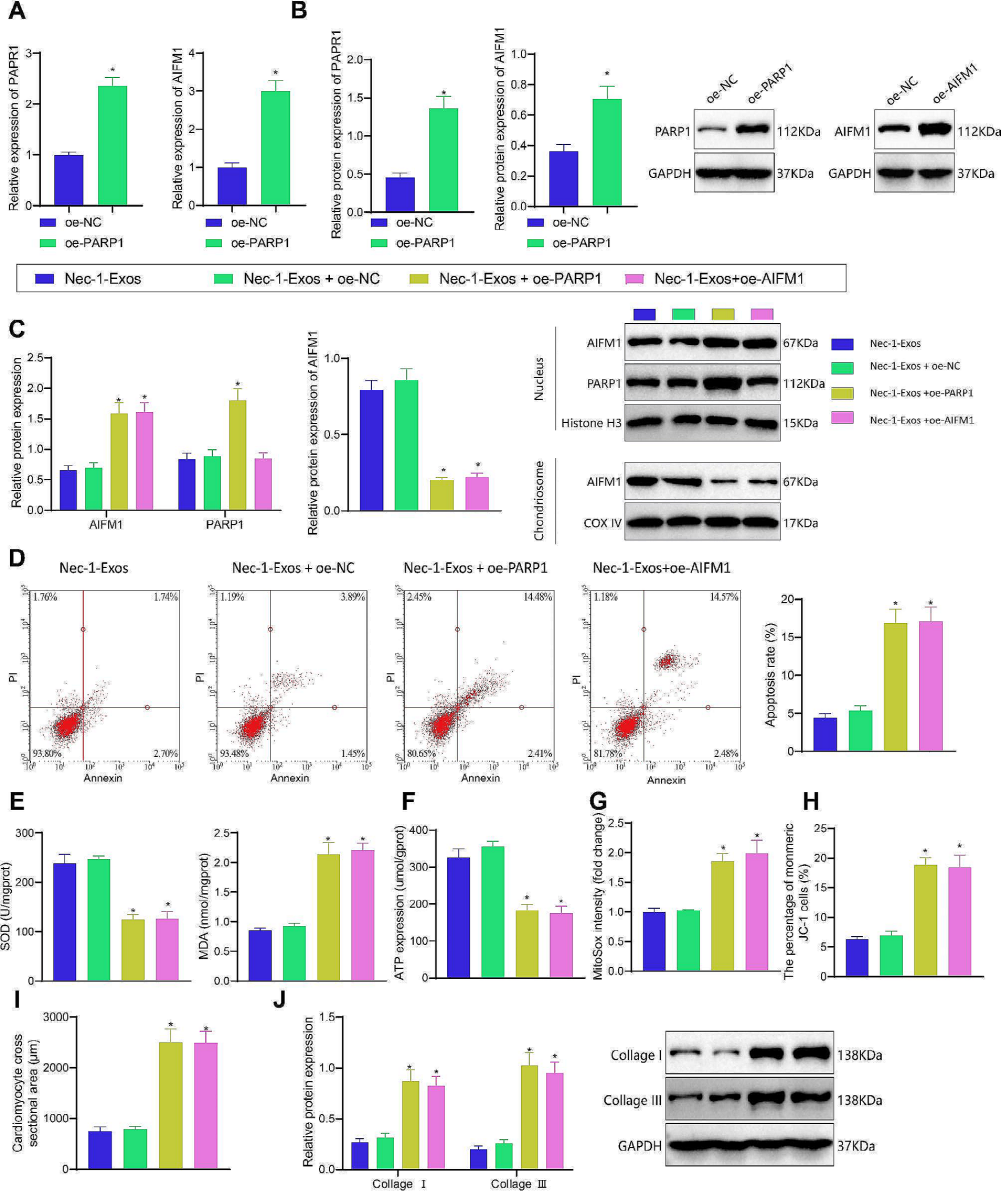
Nec-1-Exos affects oxidative stress and mitochondrial dysfunction in cardiomyocytes through PARP1/AIFM1. HF cardiomyocytes were transduced with oe-PARP1. **A**, The efficiency of PARP1 overexpression detected by RT-qPCR. **B**, The efficiency of PARP1 overexpression detected by Western blot analysis. HF cardiomyocytes were treated with Nec-1-Exos alone or combined with oe-PARP1. **C**, Western blot analysis of PARP1 and AIFM1 protein expression in the nucleus and mitochondria of each treatment group. **D**, Apoptosis of HF cardiomyocytes detected by flow cytometry. **E**, Measurement of SOD activity and MDA level in HF cardiomyocytes. **F**, Detection of mitochondrial ATP generation ability in HF cardiomyocytes. **G**, Mitochondrial ROS generation ability in HF cardiomyocytes detected by Mito-SOX. **H**, The changes in mitochondrial membrane potential detected by flow cytometry. **I**, Cardiomyocyte hypertrophy of HF cardiomyocytes detected using phalloidin-FITC staining. **J**, Protein levels of Collagen I and Collagen III in HF cardiomyocytes determined using Western blot analysis. * *p* < 0.05 vs. HF cardiomyocytes transduced with oe-NC or HF cardiomyocytes treated with Nec-1-Exos + oe-NC. Cell experiments were repeated three times

Further investigation was conducted to assess the impact of Nec-1-Exos on oxidative stress and mitochondrial function in cardiomyocytes through the PARP1/AIFM1 signaling axis. Quantitative real-time polymerase chain reaction (qRT-PCR) and Western blot were utilized to evaluate the overexpression efficiency of PARP1 and AIFM1. Compared to the oe-NC group, the oe-PARP1 group exhibited a significant increase in PARP1 expression, while the oe-AIFM1 group showed a notable increase in AIFM1 expression compared to the oe-NC group (Fig. 6A, B). Compared to the Nec-1-Exos + oe-NC group, the Nec-1-Exos + oe-PARP1 group exhibited a significant increase in nuclear PARP1 and AIFM1 expression, while mitochondrial AIFM1 expression was notably reduced. In contrast, compared to the Nec-1-Exos + oe-NC group, the Nec-1-Exos + oe-AIFM1 group showed no significant change in nuclear PARP1 expression, a significant increase in AIFM1 expression, and a significant decrease in mitochondrial AIFM1 expression (Fig. 6C). Flow cytometry results indicated that compared to the Nec-1-Exos + oe-NC group, the Nec-1-Exos + oe-PARP1 group had a significantly higher apoptosis rate, and similarly, the Nec-1-Exos + oe-AIFM1 group had a significantly increased apoptosis rate compared to the Nec-1-Exos + oe-NC group (Fig. 6D).

Results of antioxidant capacity assessments showed that compared to the Nec-1-Exos + oe-NC group, the Nec-1-Exos + oe-PARP1 group had significantly reduced cellular SOD activity and increased MDA levels, while the Nec-1-Exos + oe-AIFM1 group exhibited significantly decreased SOD activity and increased MDA levels (Fig. 6E). Mitochondrial function assessments indicated that compared to the Nec-1-Exos + oe-NC group, the Nec-1-Exos + oe-PARP1 group showed significantly decreased ATP levels and membrane potential but increased ROS levels. Similarly, the Nec-1-Exos + oe-AIFM1 group displayed significantly decreased ATP levels and membrane potential but increased ROS levels compared to the Nec-1-Exos + oe-NC group (Fig. 6F-H). Phalloidin-FITC staining results revealed that the cross-sectional area of cells significantly increased in the Nec-1-Exos + oe-PARP1 group compared to the Nec-1-Exos + oe-NC group, and similarly, the cross-sectional area significantly increased in the Nec-1-Exos + oe-AIFM1 group compared to the Nec-1-Exos + oe-NC group (Fig. 6I). Furthermore, Western blot analysis demonstrated that the expression of Collagen I and Collagen III proteins significantly increased in the Nec-1-Exos + oe-PARP1 group and the Nec-1-Exos + oe-AIFM1 group compared to the Nec-1-Exos + oe-NC group (Fig. 6J).

These results suggest that Nec-1-Exos inhibit oxidative stress and mitochondrial dysfunction in cardiomyocytes by downregulating the PARP1/AIFM1 signaling axis, and confirming AIFM1’s position downstream of the PARP1 signaling pathway.

### Nec-1-Exos inhibit the PARP1/AIFM1 axis to relieve HF in vivo

**Fig. 7:**
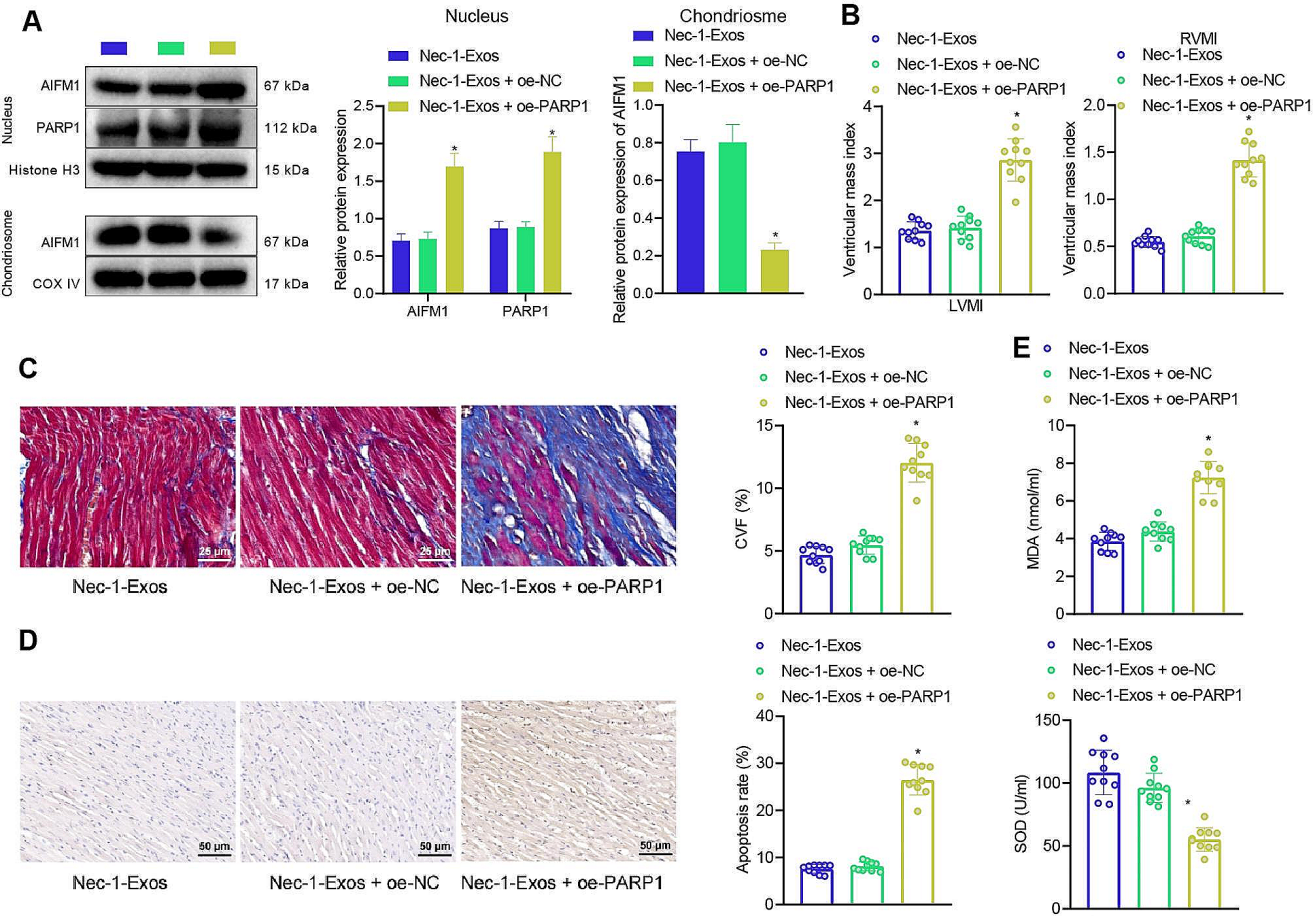
Effect of Nec-1-Exos on HF via PARP1/AIFM1 in vivo. HF rats were injected with Nec-1-Exos alone or combined with oe-PARP1 (*n* = 10). **A**, Western blot analysis of PARP1 and AIFM1 protein expression in the nuclei and mitochondria of cardiac tissue samples from each treatment group (*n* = 10). **B**, Changes of ventricular mass index in HF rats. **C**, Collagen fibers in myocardial tissues of HF rats detected using Masson staining. **D**, Apoptosis in myocardial tissues of HF rats detected using TUNEL staining. **E**, Measurement of SOD activity and MDA level in HF rats. * *p* < 0.05 vs. HF rats injected with Nec-1-Exos + oe-NC

The effect of Nec-1-Exos-mediated PARP1/AIFM1 axis on HF was further verified in the rat models. Western blot analysis presented that the expression of AIFM1 and PARP1 increased in the nucleus, and AIFM1 expression decreased in the mitochondria of HF rats injected with Nec-1-Exos + oe-PARP1 (Fig. 7A). Ultrasound diagnostic instrument showed that IVSD, LVEDD, LVESD, and LVPWD increased, while LVEF and FS decreased in HF rats injected with Nec-1-Exos + oe-PARP1 (Table S6).

Hemodynamics exhibited an increase in LVEDP and declines in LVSP, +dp/dt, and -dp/dt in HF rats injected with Nec-1-Exos + oe-PARP1 (Table S7). The ventricular mass index test also found that LVMI and RVMI increased in HF rats injected with Nec-1-Exos + oe-PARP1 (Fig. 7B).

Masson staining and TUNEL staining displayed increased CVF and apoptosis in HF rats injected with Nec-1-Exos + oe-PARP1 (Fig. 7C, D). Antioxidant index test showed that SOD activity was reduced, and the MDA level was elevated in the heart tissues of HF rats injected with Nec-1-Exos + oe-PARP1 (Fig. 7E).

The obtained data suggested that Nec-1-Exos could alleviate HF in rats by inhibiting PARP1/AIFM1 axis.

## Discussion

HF is a chronic and progressive clinical syndrome induced by structural or functional cardiac abnormalities [2]. In recent studies, modulation of mitochondrial dysfunction and oxidative stress is a potential way for HF treatment [6]. As the specific mechanisms by which exosomes affect the mitochondrial dysfunction and oxidative stress in HF remain to be excavated, the objective of our study was to investigate the effect of exosomes-encapsulated Nec-1 from iPSCs, in oxidative stress and the mitochondrial dysfunction in HF and their inner mechanisms. Data obtained in our study demonstrated that Nec-1 shuttled by iPSCs-derived exosomes exerted inhibitory properties in oxidative stress and the mitochondrial dysfunction to attenuate HF through the PARP1/AIFM1 axis both in vitro and in vivo.

The finding in the present study demonstrated that iPSCs-derived exosomes could deliver Nec-1 into cardiomyocytes. The iPSCs have been utilized in the treatment of HF due to their self-renewal and differentiation potentiality into cardiomyocytes [25]. Existing literatures have validated that iPSCs-secreted exosomes function as a potential biological source for future therapy for HF [14, 15]. Exosomes contain cargoes, such as coding and noncoding RNAs, proteins, and signaling molecules, which can be delivered into recipient cells, inducing an intracellular response [26]. Moreover, the obtained data suggested that iPSCs-derived exosomes-loaded Nec-1 inhibits oxidative stress and mitochondrial dysfunction in HF by increasing ATP and membrane potential and reducing ROS, Collagen I, and Collagen III. Mitochondrial dysfunction is considered a hallmark of HF and a main cause of oxidative stress, which in turn leads to the damage of cellular components, including mitochondria themselves, resulting in a vicious circle [27]. Oxidative stress is also implicated in the development of HF [28]. Oxidative damage contributes to the damage of the mitochondrial electron transport chain and leads to bioenergy dysfunction by reducing ATP production and accumulation of ROS [29]. It has been demonstrated that reduction of excessive ROS is helpful in alleviating HF [6]. Similarly, iPSC-derived exosomes could reduce oxidative stress to relieve myocardial injury [30]. The role of exosomes in HF by modulating oxidative hemostasis has also been demonstrated [31].

In addition, Nec-1 suppressed myocardial contractile dysfunction by inhibiting the production of ROS [19]. Nec-1 may inhibit cardiomyocyte death and reduce infarct size by affecting mitochondrial permeability [32]. Nec-1 could attenuate HF by blocking necroptosis via reduction of mitochondrial ROS and elevation of ATP [18], which is consistent with our findings.

Existing literatures have verified that iPSC-derived exosomes might provide a potential cell-free treatment for HF [14, 15]. Moreover, chemotherapeutic drugs, protein peptides, miRNAs, etc. could be effectively introduced into exosomes through electroporation, which exhibit a good therapeutic effect on various diseases [33]. Accumulating evidences have proved that Nec-1 might inhibit myocardial cell death by affecting mitochondrial permeability and reduce the infarct size [32, 34]. Furthermore, it can also be concluded that iPSCs-exosomes carrying Nec-1 downregulated PARP1 and inhibited AIFM1 nuclear translocation to alleviate HF by suppressing cardiomyocyte apoptosis. During the HF process, cardiac cells start to die through apoptosis and/or oxidative injury, resulting in myocardial cellular necrosis [35].

Although this study found that Nec-1 may improve myocardial cell oxidative stress and mitochondrial function by downregulating PARP1 expression and inhibiting AIFM1 nuclear translocation, ultimately alleviating heart failure in rats, previous research has also demonstrated that RIPK1 can interact with PARP1 to suppress oxidative stress or necrosis [36, 37]. This suggests that the protective effect of Nec-1 on myocardial cells following PARP1 downregulation may involve other downstream signaling pathways, including RIPK1. Subsequent studies will prioritize investigating the role of RIPK1 in this process.

Prior studies have shown that exosomes secreted by iPSCs contain a complex array of contents [38, 39]. Some research indicates that miR-100-5p present in iPSC-secreted exosomes can exert cellular protective effects by regulating the dynamic balance of intracellular Ca2+ [40]. Other studies have suggested that iPSC-derived exosomes may contain proteins that clear intracellular ROS, thereby reducing intracellular ROS levels and providing cellular protection [41]. Overall, our analysis suggests that there may be unidentified compounds in iPSC-secreted exosomes that potentially protect the heart, representing a limitation of this study. However, we believe that this limitation does not undermine the main conclusions and findings of our current research. Moving forward, we plan to further investigate the presence and regulatory mechanisms of other potentially cardioprotective substances in iPSC-secreted exosomes, if conditions permit.

Cardiomyocyte apoptosis is one of the most significant pathophysiological processes of cardiomyocyte repair and overall compensation following HF [3]. Meanwhile, multiple studies have demonstrated that Nec-1 downregulates the expression of PARP1 and AIFM1 [42, 43]. Multiple studies demonstrated that Nec could downregulate the expression of PARP1 and AIFM1 through CTD database analysis [42, 43]. PARP1 is an important Caspase-independent cell death activator, which directly or indirectly regulated the translocation of AIFM1 in cardiomyocytes under oxidative stress, induced mitochondrial dysfunction, and promoted the pathway of mitochondrial cell death [44, 45]. Therefore, Nec-1 might be involved in the regulation of oxidative stress and mitochondrial dysfunction in cardiomyocytes through PARP1 and AIFM1. It is known that PARP1 is implicated in HF [46]. Oxidative stress leads to DNA breaks activating PARP1 to induce HF, and inhibition of the PARP enzyme could offer a promising new therapeutic approach to prevent the onset of HF [47]. PARP1, as an important caspase independent cell death activator, which directly or indirectly regulates the translocation of AIFM1 in cardiomyocytes under oxidative stress, induces mitochondrial dysfunction and promotes the mitochondrial cell death pathway [44, 45]. AIFM1 is located on chromosome X and encodes for apoptosis-inducing factor, a mitochondrial flavoprotein involved in caspase-independent cell death [48]. The roles of AIFM1 in the regulation of apoptosis, ROS generation, and ATP production have also been investigated [49]. In the current study, both in vitro and in vivo experiments revealed that Nec-1 shuttled by iPSCs-secreted exosomes inhibited oxidative stress and mitochondrial dysfunction to relieve HF via downregulation of PARP1/AIFM1 axis.

**Fig. 8:**
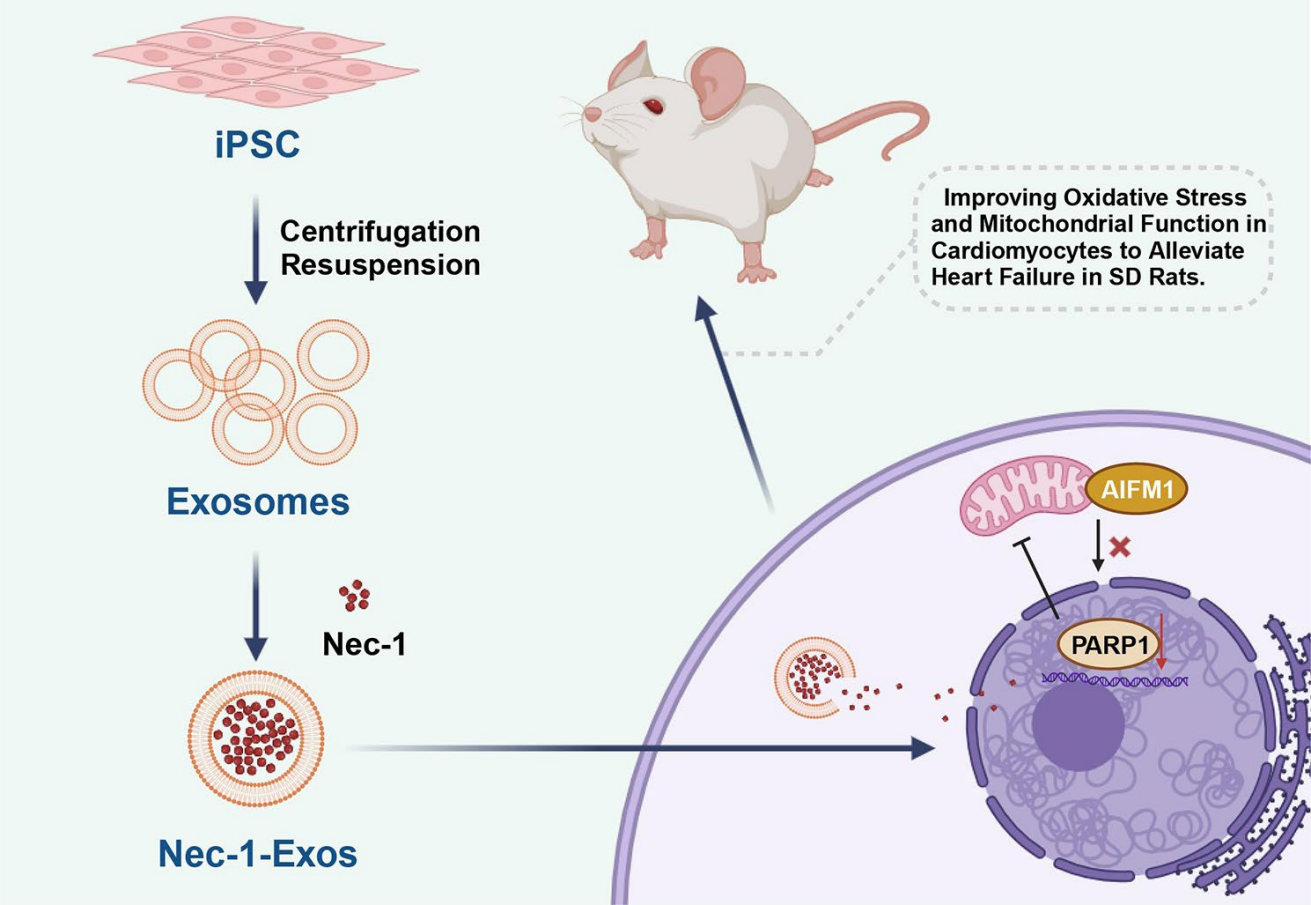
Molecular mechanism of iPSCs-derived exosomes-loaded Nec-1 mediating PARP1/AIFM1 pathway to affect oxidative stress and mitochondrial dysfunction of cardiomyocytes in HF

In conclusion, our study verified that the transfer of Nec-1 via iPSCs-secreted exosomes altered PARP1 and AIFM1 expression, which led to the alleviation of HF (Fig. 8). Our findings provide novel insights for the development of potential therapeutic strategies for inhibiting oxidative stress and mitochondrial dysfunction to attenuate HF. Due to the limited known researches, the roles of Nec-1 shuttled by iPSCs-derived exosomes, PARP1, AIFM1, as well as their interaction in oxidative stress and mitochondrial dysfunction in the progression of HF should be more clearly investigated.

### Electronic supplementary material

Below is the link to the electronic supplementary material.

Supplementary Material 1

Supplementary Material 2. Fig. S1 Representative in vitro fluorescence image showing the distribution of DiR-labeled exosomes in various organs of rats

Supplementary Material 3

## Data Availability

The datasets used and/or analyzed during the current study are available from the corresponding author on reasonable request.

## Abbreviations

HF: Heart failure
iPSCs: induced pluripotent stem cells
MSCs: mesenchymal stem cells
Nec-1: Necrostatin-1
SD: Sprague–Dawley
TAC: trans-ascending aortic constriction
LVEF: left-ventricular ejection fraction
NC: negative control
LVPWD: left-ventricular posterior wall dimension
IVSD: interventricular septal dimension
LVEDD: left-ventricular end diastolic dimension
FS: fraction shortening
PBS: phosphate buffered saline
TEM: transmission electron microscopy
DLS: Dynamic Light Scattering
