# Supplementary figures and images for: Unlocking cardioprotection: iPSC exosomes deliver Nec-1 to target PARP1/AIFM1 axis, alleviating HF oxidative stress and mitochondrial dysfunction
Source: J Transl Med. 2024 Jul 26;22:681. doi: 10.1186/s12967-024-05204-9 (PMC11282728; doi:10.1186/s12967-024-05204-9)

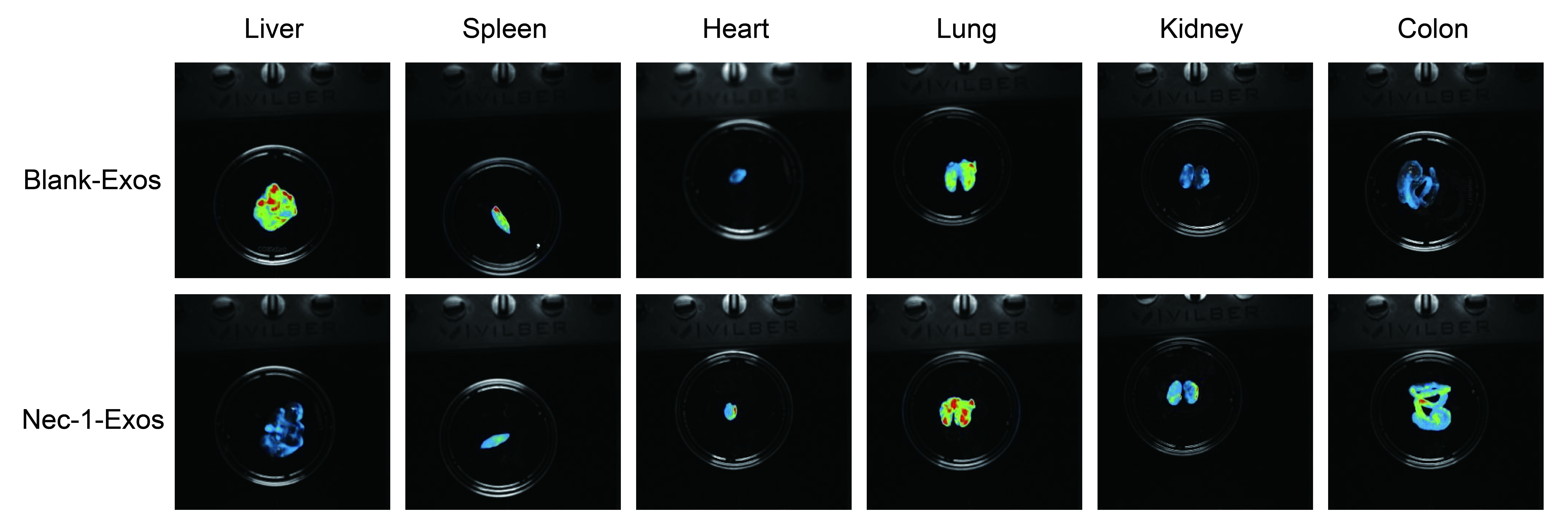

Supplement: Supplementary file 2 — Supplementary Material 2. Fig. S1 Representative in vitro fluorescence image showing the distribution of DiR-labeled exosomes in various organs of rats [file 12967_2024_5204_MOESM2_ESM.jpg]
