# Supplementary material for: Unlocking cardioprotection: iPSC exosomes deliver Nec-1 to target PARP1/AIFM1 axis, alleviating HF oxidative stress and mitochondrial dysfunction
Source: J Transl Med. 2024 Jul 26;22:681. doi: 10.1186/s12967-024-05204-9 (PMC11282728; doi:10.1186/s12967-024-05204-9)
Supplement: Supplementary file 3 — Supplementary Material 3 [file 12967_2024_5204_MOESM3_ESM.docx]

**Table S1** Primer sequences of RT-qPCR

| Genes | Primer sequences (5’-3’) |
| --- | --- |
| PARP1 | Forward: 5’-TACCATCTGGAGAGTCCGCA-3’ |
|  | Reverse: 5’-CCTTTCGGCTGGGATTCTGT-3’ |
| AIFM1 | Forward: 5’-CGGCGGTGTGTGAAAAGAAA-3’ |
|  | Reverse: 5’-ATTTTGCCCCCTGATGGACC-3’ |
| GAPDH | Forward: 5’-GCATCTTCTTGTGCAGTGCC-3’ |
|  | Reverse: 5’-GATGGTGATGGGTTTCCCGT-3’ |

Note: GAPDH, glyceraldehyde-3-phosphate dehydrogenase; PARP1, poly(ADP-ribose) polymerase 1; AIFM1, apoptosis inducing factor mitochondria associated 1; RT-qPCR, reverse transcription quantitative polymerase chain reaction.

**Table S2** Echocardiographic results of rats in each group (mean ± standard deviation)

| Group | IVSD (mm) | LVEDD (mm) | LVESD (mm) | LVPWD (mm) | LVEF (%) | FS (%) |
| --- | --- | --- | --- | --- | --- | --- |
| Normal | 1.25 ± 0.16 | 3.24 ± 0.38 | 2.38 ± 0.33 | 0.89 ± 0.14 | 78.16 ± 8.96 | 29.76 ± 3.28 |
| Sham | 1.47 ± 0.19 | 3.58 ± 0.44 | 2.57 ± 0.44 | 0.92 ± 0.12 | 83.01 ± 9.14 | 32.49 ± 2.54 |
| Model | 3.68 ± 0.45* | 6.89 ± 0.76* | 5.06 ± 0.58* | 1.79 ± 0.23* | 36.15 ± 4.58* | 16.03 ± 1.24* |

Note: Data comparison was analyzed by one-way ANOVA. * *p* < 0.05 *vs.* sham-operated rats. IVSD, interventricular septal dimension; LVEDD, left-ventricular end diastolic dimension; LVESD, left-ventricular end systolic diameter; LVEF, left-ventricular ejection fraction; FS, fraction shortening.

**Table S3** Hemodynamic indexes of rats in each group (mean ± standard deviation)

| Group | LVEDP (mm Hg) | LVSP (mm Hg) | +dp/dt (mm Hg/s) | -dp/dt (mm Hg/s) |
| --- | --- | --- | --- | --- |
| Normal | 8.24 ± 0.96 | 158.49 ± 17.86 | 4501.21 ± 542.34 | -2245.15 ± 201.66 |
| Sham | 7.63±0.91 | 163.25 ± 13.28 | 4987.86 ± 504.85 | -2058.99 ± 236.78 |
| Model | 28.46 ± 3.26* | 63.06 ± 5.25* | 1426.25 ± 165.22* | -869.59 ± 96.89* |

Note: Data comparison was analyzed by one-way ANOVA. * *p* < 0.05 *vs.* sham-operated rats. LVEDP, left-ventricular end diastolic pressure; LVSP, left-ventricular systolic pressure.

**Table S4** Echocardiographic results of HF rats injected with blank-Exos or Nec-1-Exos (mean ± standard deviation)

| Group | IVSD (mm) | LVEDD (mm) | LVESD (mm) | LVPWD (mm) | LVEF (%) | FS (%) |
| --- | --- | --- | --- | --- | --- | --- |
| Model | 3.68 ± 0.45* | 6.89 ± 0.76* | 5.06 ± 0.58* | 1.79 ± 0.23* | 36.15 ± 4.58* | 16.03 ± 1.24* |
| Nec-1 | 2.24 ± 0.27* | 5.17± 0.52* | 2.86 ± 0.34* | 2.49 ± 0.32* | 62.79 ± 8.13* | 26.35± 3.64* |
| blank-Exos | 2.01 ± 0.25* | 4.87 ± 0.63* | 3.05 ± 0.24* | 2.57 ± 0.23* | 60.14 ± 5.57* | 28.49 ± 3.47* |
| Nec-1-Exos | 1.19 ± 0.17#& | 2.56 ± 0.34#& | 1.18 ± 0.16#& | 3.18 ± 0.44#& | 91.49 ± 8.22#& | 40.98 ± 5.09#& |

Note: Data comparison was analyzed by one-way ANOVA. * *p* < 0.05 *vs.* IF rats. # *p* < 0.05 *vs.* IF rats injected with blank-Exos. & *p* < 0.05 *vs.* IF rats injected with Nec-1.

**Table S5** Hemodynamic indexes of HF rats injected with blank-Exos or Nec-1-Exos (mean ± standard deviation)

| Group | LVEDP (mm Hg) | LVSP (mm Hg) | +dp/dt (mm Hg/s) | -dp/dt (mm Hg/s) |
| --- | --- | --- | --- | --- |
| Model | 28.46 ± 3.26* | 63.06 ± 5.25* | 1426.25 ± 165.22* | -869.59 ± 96.89* |
| Nec-1 | 15.24 ± 1.61* | 101.67 ± 11.97* | 2416.73 ± 249.59* | -1521.67 ± 208.24* |
| blank-Exos | 16.89 ± 2.36* | 106.75 ± 12.75* | 2697.18 ± 302.14* | -1631.08 ± 185.94* |
| Nec-1-Exos | 7.04 ± 0.97#& | 163.42 ± 18.94#& | 4767.38 ± 518.33#& | -2279.36 ± 168.99#& |

Note: Data comparison was analyzed by one-way ANOVA. * *p* < 0.05 *vs.* IF rats. # *p* < 0.05 *vs.* IF rats injected with blank-Exos. & *p* < 0.05 *vs.* IF rats injected with Nec-1. LVEDP, left-ventricular end diastolic pressure; LVSP, left-ventricular systolic pressure.

**Table S6** Echocardiographic results of HF rats injected with Nec-1-Exos and/or oe-PARP1 (mean ± standard deviation)

| Group | IVSD (mm) | LVEDD (mm) | LVESD (mm) | LVPWD (mm) | LVEF (%) | FS (%) |
| --- | --- | --- | --- | --- | --- | --- |
| Nec-1-Exos | 1.19 ± 0.17 | 2.56 ± 0.34 | 1.18 ± 0.16 | 3.18 ± 0.44 | 91.49 ± 8.22 | 40.98 ± 5.09 |
| Nec-1-Exos + oe-NC | 1.38 ± 0.21 | 3.02 ± 0.38 | 1.36 ± 0.17 | 4.86 ± 0.58 | 95.14 ± 8.25 | 35.14 ± 4.31 |
| Nec-1-Exos + oe-PARP1 | 3.25 ± 0.26* | 7.14 ± 0.62* | 4.89 ± 0.55* | 4.71 ± 0.63* | 32.06 ± 4.31* | 11.02 ± 1.61* |

Note: Data comparison was analyzed by one-way ANOVA. * *p* < 0.05 *vs.* IF rats injected withNec-1-Exos + oe-NC. n = 10. IVSD, interventricular septal dimension; LVEDD, left-ventricular end diastolic dimension; LVESD, left-ventricular end systolic diameter; LVEF, left-ventricular ejection fraction; FS, fraction shortening; NC, negative control; oe-, overexpression.

**Table S7** Hemodynamic indexes of HF rats injected with Nec-1-Exos and/or oe-PARP1 (mean ± standard deviation)

| Group | LVEDP (mm Hg) | LVSP (mm Hg) | +dp/dt (mm Hg/s) | -dp/dt (mm Hg/s) |
| --- | --- | --- | --- | --- |
| Nec-1-Exos | 7.04 ± 0.97 | 163.42 ± 18.94 | 4767.38 ± 518.33 | -2279.36 ± 168.99 |
| Nec-1-Exos + oe-NC | 7.29 ± 0.92 | 154.22 ± 17.86 | 4887.29 ± 436.11 | -2088.16 ± 190.30 |
| Nec-1-Exos + oe-PARP1 | 24.79 ± 3.14* | 49.14 ± 5.37* | 1532.02 ± 164.79* | -823.28 ± 101.03* |

Note: Data comparison was analyzed by one-way ANOVA. * *p* < 0.05 *vs.* IF rats injected with Nec-1-Exos + oe-NC. n = 10. LVEDP, left-ventricular end diastolic pressure; LVSP, left-ventricular systolic pressure.
