# Supplementary material for: Unlocking cardioprotection: iPSC exosomes deliver Nec-1 to target PARP1/AIFM1 axis, alleviating HF oxidative stress and mitochondrial dysfunction
Source: J Transl Med. 2024 Jul 26;22:681. doi: 10.1186/s12967-024-05204-9 (PMC11282728; doi:10.1186/s12967-024-05204-9)
Supplement: Supplementary file 1 — Supplementary Material 1 [file 12967_2024_5204_MOESM1_ESM.docx]

Reviewer reports:

Reviewer #1: This paper studied the cardio-protective effects of exosome-delivered Nec-1 on hypertrophic cardiac tissue and cardiomyocytes, and exogenous Nec-1 could significantly improve apoptosis and oxidative stress induced by hypertrophic stimulus. The authors used exosomes derived from iPSC to carry Nec-1 into cardiomyocytes, exploring the molecular mechanism of Nec-1. The whole paper is well-written, and the experiments seem well-described, but some points need to be further addressed.

Major points:

1. It is well known that Nec-1 is a selective inhibitor of RIP1 and could inhibit necroptosis by repressing RIP1 kinase activity and RIP1-RIP3 interaction. In this paper, the author demonstrated that Nec-1 could downregulate the nucleus expression of PARP1. Ki-Hong Jang, et al (PMID: 28993228) and S Jouan-Lanhouet, et al (PMID: 22814620) have proven that RIPK1 could interact with PARP1, consequently inhibiting oxidative stress or necroptosis. Taken together, RIPK1 might also account for Nec-1's cardioprotective role, thus the role of RIPK1 should be explored and discussed.

**Response:** Thank you for your review. In order to further elaborate on the findings of our study, the discussion section has been enriched with the following content: While our research suggests that Nec-1 may improve myocardial cell oxidative stress and mitochondrial function by downregulating PARP1 expression and inhibiting AIFM1 nuclear translocation, ultimately alleviating heart failure in rats, previous studies have indicated an interaction between RIPK1 and PARP1, leading to the inhibition of oxidative stress or necrosis (PMID: 22814620, PMID: 28993228). This implication suggests that after the downregulation of PARP1 by Nec-1, additional downstream signaling pathways, including RIPK1, might play a role in protecting myocardial cells. In future studies, we plan to prioritize the investigation of the role of RIPK1 in this context.

2. From Fig.3 and Fig.4, it is interesting to find that the Blank-Exos group showed decreased cardiac remodeling, apoptotic level, and oxidative stress compared with the HF group. These data indicated that Exos solely improves cardiac injury, the authors should explain the potential reasons.

**Response:** To address this phenomenon, the discussion section has been enriched with the following insights: Previous studies have indicated that exosomes secreted by iPSCs contain complex cargo components (PMID: 36902008, PMID: 32117968). Research has suggested that miR-100-5p found in iPSC-derived exosomes may exert cellular protective effects by regulating the dynamic balance of intracellular Ca2+ levels (PMID: 37775801). Additionally, some studies have revealed the presence of proteins in iPSC-derived exosomes that could potentially eliminate intracellular reactive oxygen species (ROS), thereby reducing cellular ROS levels and providing cellular protection (PMID: 30811771). Through comprehensive analysis, it is speculated that there may be unidentified compounds present in iPSC-secreted exosomes that could potentially offer protective effects to the heart, representing a limitation of this study. Nevertheless, it is believed that this limitation does not undermine the primary conclusions and findings of the current research. Future investigations, as circumstances allow, will focus on exploring the existence of other potentially cardioprotective substances in iPSC-derived exosomes and elucidating their underlying regulatory mechanisms.

3. This study showed that the increased Nec-1 inhibited nucleus PARP1 level and AIFM1 nuclear translocation and overexpression PARP1 also led to an increased AIFM in the nucleus. STRING database exhibited that there was a regulatory relationship between PARP1 and AIFM1. It would be more reliable to test the interacting effect between PARP1 and AIFM.

**Response:** In Figure 5G, we have conducted a Co-IP assay to investigate the interaction between PARP1 and AIFM1. The results show that AIFM1 protein was detected in the complex pulled down by the PARP1 antibody, indicating the presence of interaction between PARP1 and AIFM1.

Minor points：

1. In Fig2E, the author examined the protein levels of RIPK1 and RIPK3 to show that isolated Nec-1 exosomes were indeed transferred into cardiomyocytes. The levels of Nec-1 should also be tested to confirm exosomes inside.

**Response:** In Figure 2F, we have supplemented the detection of Nec-1 levels in cardiomyocytes using LC-MS/MS. The results show a significant increase in the Nec-1 content in cardiomyocytes of the Nec-1-exos group compared to the Blank-exos group, indicating successful delivery of Nec-1 into cardiomyocytes.

2. There are plenty of errors in Figures, including confusing layouts and labels. For example, In Fig1B, the scale label is missing in the model group; in Fig.5D, there are four groups but only three indicated labels.

**Response:** We have revised the figures to correct the errors in layouts and labels as pointed out. This includes adding the missing scale label in Fig.1B and correcting the group labels in Fig.5D to ensure clarity and accuracy.

3. Fig.6 and Fig.7 illustrate which parts of the nucleus and mitochondria are unknown regarding PARP1 and AIFM1 levels.

**Response:** After reviewing the images, we identified that the organelle names corresponding to WB were not clearly labeled in Figure 6C and Figure 7A. Relevant information has been added for clarification.

**Reviewer #2:**

1-Code of ethical approval is not mentioned.

**Response:** Thank you for you review. We have now included the code of ethical approval within the manuscript to comply with ethical reporting standards.

2-It is not mentioned how the animals were disposed of in a hygienical and ethical manner at the end of the study.

**Response:** We have added information regarding the disposal method after the conclusion of the experiment, which involves euthanizing the mice by cervical dislocation at the end of the experiment.

3-You discussed your results with results of others in the result chapter in page 5 under the title (Successful construction of exosomes loaded with Nec-1) and in page 18 under the title (Nec-1-Exos downregulate PARP1 in the nucleus and inhibit AIFM1 nuclear translocation). Comparison of the results of your study with the results of others better to be in the discussion and not in the result chapter.

**Response:** Based on your suggestion, the comparison with other studies has been moved to the Discussion section.

**Reviewer #3:** This manuscript deals with "Unlocking cardioprotection: iPSC exosomes deliver nec-1 to target PARP1/AIFM1 axis, alleviating HF oxidative stress and mitochondrial dysfunction". Heart failure (HF) is characterized by oxidative stress and mitochondrial dysfunction. This study investigates the therapeutic potential of Necrostatin-1 (Nec-1) delivered through exosomes derived from induced pluripotent stem cells (iPSCs) to address these pathologies in HF. Finally the author get the conclusion that iPSCs-derived exosomes carrying Nec-1 effectively attenuate oxidative stress and mitochondrial dysfunction in HF by targeting the PARP1/ AIFM1 axis. This study proposes a promising therapeutic strategy for HF management, highlighting the potential of exosome-mediated drug delivery. Thus, the research has certain innovation and application value. Unfortunately, there are still some deficiencies to be improved:

1. Manuscript needs considerable editing by a native English speaker.

**Response:** Thank you for your review. The manuscript has undergone thorough editing by a native English speaker to improve clarity and readability.

2. Suggest rearranging some figures without leaving any blank space, such as Figure 3 and 5.

**Response:** Figures 3 and 5 have been rearranged to eliminate any blank spaces, improving the presentation and flow of data.

3. The data shows that both Exos and Nec-1 have an improvement effect on HF, and it is recommended to add a Nec-1 administration group.

**Response:** In Figure 3, we added a treatment group with Nec-1 administered alone. The results show that compared to the Nec-1 group, the Nec-1-Exos group exhibits a further significant reduction in cell apoptosis (Figure 3A). Additionally, the Nec-1-Exos group demonstrates a further significant increase in SOD activity and a decrease in MDA levels compared to the Nec-1 group (Figure 3B). Furthermore, compared to the Nec-1 group, the Nec-1-Exos group displays a further significant increase in ATP and membrane potential, along with a further significant decrease in ROS levels (Figure 3C-E). Cell cross-sectional area is also significantly reduced in the Nec-1-Exos group compared to the Nec-1 group (Figure 3F). Collagen I and Collagen III protein expression is further significantly reduced in the Nec-1-Exos group compared to the Nec-1 group (Figure 3G). The indices in the Nec-1-Exos group show significant improvement compared to the Nec-1 group (Supplementary Table 4). Hemodynamic measurements reveal that compared to the Model group, left ventricular end-diastolic pressure (LVEDP) is significantly reduced, while left ventricular systolic pressure (LVSP), maximum rate of pressure rise in the left ventricle (+dp/dt), and maximum rate of pressure fall in the left ventricle (-dp/dt) are significantly increased in the Blank-Exos group. Further improvements are observed in the Nec-1-Exos group compared to the Blank-Exos group, and these improvements are even more pronounced than those in the Nec-1 group (Supplementary Table 5). Left and right ventricular mass indices (LVMI and RVMI) are significantly decreased in the Nec-1-Exos group compared to the Nec-1 group (Figure 4A). Masson's trichrome staining shows a further significant reduction in collagen volume fraction (CVF) in rats treated with Nec-1-Exos compared to those treated with Nec-1 alone (Figure 4B). TUNEL immunohistochemical staining indicates a further significant decrease in cell apoptosis in the Nec-1-Exos group compared to the Nec-1 group (Figure 4C). Antioxidant index measurements show a further significant increase in SOD activity and a significant decrease in MDA levels in the Nec-1-Exos group compared to the Nec-1 group (Figure 4D). The above results indicate that the effectiveness of Nec-1-loaded Exos is superior to that of Nec-1 administered alone.

4. The western blotting result group in Figure 5 is missing one group.

**Response:** The missing group in Figure 5 has been corrected.

5. Suggest adding overexpression group of AIFM1 for detection.

**Response:** In Figure 6, we have added an oe-AIFM1-related treatment group. The results indicate that compared to the oe-NC group, the oe-AIFM1 group shows a significant increase in AIFM1 expression (Figure 6A-B). In comparison to the Nec-1-Exos+oe-NC group, no significant change was observed in nuclear PARP1 expression in the Nec-1-Exos+oe-AIFM1 group, while AIFM1 expression significantly increased, and mitochondrial AIFM1 expression significantly decreased (Figure 6C). The apoptosis rate in the Nec-1-Exos+oe-AIFM1 group significantly increased compared to the Nec-1-Exos+oe-NC group (Figure 6D). Furthermore, the SOD activity in the Nec-1-Exos+oe-AIFM1 group significantly decreased, and MDA levels increased significantly compared to the Nec-1-Exos+oe-NC group (Figure 6E). Additionally, ATP levels and membrane potential significantly decreased, while ROS levels increased significantly in the Nec-1-Exos+oe-AIFM1 group compared to the Nec-1-Exos+oe-NC group (Figure 6F-H). The cross-sectional area of cells in the Nec-1-Exos+oe-AIFM1 group significantly increased compared to the Nec-1-Exos+oe-NC group (Figure 6I). Moreover, the expression of Collagen I and Collagen III proteins significantly increased in the cells of the Nec-1-Exos+oe-AIFM1 group compared to the Nec-1-Exos+oe-NC group (Figure 6J).
